# ELF5 isoform expression is tissue-specific and significantly altered in cancer

**DOI:** 10.1186/s13058-015-0666-0

**Published:** 2016-01-07

**Authors:** Catherine L. Piggin, Daniel L. Roden, David Gallego-Ortega, Heather J. Lee, Samantha R. Oakes, Christopher J. Ormandy

**Affiliations:** Cancer Division, Garvan Institute of Medical Research/The Kinghorn Cancer Centre, Sydney, NSW 2010 Australia; Babraham Institute, Cambridge, CB22 3AT UK

**Keywords:** ELF5, ETS transcription factors, Isoforms, Transcript variants, Splicing, Cancer

## Abstract

**Background:**

E74-like factor 5 (ELF5) is an epithelial-specific member of the E26 transforming sequence (ETS) transcription factor family and a critical regulator of cell fate in the placenta, pulmonary bronchi, and milk-producing alveoli of the mammary gland. ELF5 also plays key roles in malignancy, particularly in basal-like and endocrine-resistant forms of breast cancer. Almost all genes undergo alternative transcription or splicing, which increases the diversity of protein structure and function. Although ELF5 has multiple isoforms, this has not been considered in previous studies of ELF5 function.

**Methods:**

RNA-sequencing data for 6757 samples from The Cancer Genome Atlas were analyzed to characterize ELF5 isoform expression in multiple normal tissues and cancers. Extensive in vitro analysis of ELF5 isoforms, including a 116-gene quantitative polymerase chain reaction panel, was performed in breast cancer cell lines.

**Results:**

ELF5 isoform expression was found to be tissue-specific due to alternative promoter use but altered in multiple cancer types. The normal breast expressed one main isoform, while in breast cancer there were subtype-specific alterations in expression. Expression of other ETS factors was also significantly altered in breast cancer, with the basal-like subtype demonstrating a distinct ETS expression profile. In vitro inducible expression of the full-length isoforms 1 and 2, as well as isoform 3 (lacking the Pointed domain) had similar phenotypic and transcriptional effects.

**Conclusions:**

Alternative promoter use, conferring differential regulatory responses, is the main mechanism governing ELF5 action rather than differential transcriptional activity of the isoforms. This understanding of expression and function at the isoform level is a vital first step in realizing the potential of transcription factors such as ELF5 as prognostic markers or therapeutic targets in cancer.

**Electronic supplementary material:**

The online version of this article (doi:10.1186/s13058-015-0666-0) contains supplementary material, which is available to authorized users.

## Background

Transcription factors are the integrators of multiple signaling pathways, converting internal and external stimuli into changes in gene expression. Through this role, the evolutionarily conserved E26 transforming sequence (ETS) transcription factor family controls fundamental cellular processes such as proliferation, differentiation, and apoptosis [1]. The 28 members of the human ETS family are characterized by an ETS DNA-binding domain that recognizes a core GGAA/T motif. Additional specificity of ETS domain binding is conferred by the amino acids surrounding the key residues, as well as by posttranslational modifications and interactions with other proteins [2, 3]. Given the vital cellular processes regulated by ETS transcription factors, it is not surprising that they have also been identified as significant contributors to tumorigenesis [4].

E74-like factor 5 (ELF5) is an epithelial-specific member of the ETS transcription factor family [5, 6]. In addition to the ETS domain, the full-length ELF5 protein contains an N-terminal Pointed (PNT) domain (83 amino acids) that is similar to the evolutionarily conserved sterile alpha motif (SAM) domain. In humans, the SMART database [7] identifies 96 SAM/PNT domain-containing proteins, 11 of which are ETS family members. SAM domains have diverse functions, including protein–protein interactions, polymerization, kinase docking, RNA binding, and lipid molecule interactions [8–11]. The ELF5 PNT domain has been shown to have strong transactivation activity [12]; however, the mechanisms underlying this activity (for example, protein–protein interactions or posttranslational modifications) are unknown.

A critical function of ELF5 is the regulation of cell fate, beginning with specification of the trophectoderm in the blastocyst [13]. Correct spatial and temporal ELF5 expression is also important for normal development of the embryonic lung [14]. In the mammary gland, prolactin- and progesterone-driven ELF5 expression during pregnancy directs the development of the luminal progenitor cells into estrogen receptor-α (ER)- and progesterone receptor (PR)-negative milk-producing cells [15]. In normal human tissues, ELF5 is reported to be expressed in the kidney, prostate, lung, mammary gland, salivary gland, placenta, and stomach [5, 6, 16].

More recently, there has been increasing interest in the role of ELF5 in cancer. ETS factors are frequently deregulated in cancer through diverse mechanisms, including gene fusions, alterations in localization and/or activity, amplifications, increased expression, and (less commonly described) decreased expression [4]. ELF5 was originally described as a tumor suppressor [5]; however, the role of this protein in cancer is complex and context-dependent. In prostate cancer, for example, ELF5 has been shown to inhibit transforming growth factor (TGF)-β-driven epithelial–mesenchymal transition by blocking phosphorylation of the TGF-β effector protein SMAD3 [17]. Conversely, *ELF5* mRNA has been shown to be upregulated in a cell line model of prostate cancer progression involving acquisition of androgen independence [18]. Bladder and kidney carcinoma have been associated with loss of ELF5 expression at the protein and RNA levels [19, 20], whereas in endometrial carcinoma *ELF5* upregulation is associated with higher disease stage [21]. *ELF5* gene rearrangements have been described in several lung cancer cell lines [5], and the authors of a recent case study described a *ZFPM2-ELF5* fusion gene in multicystic mesothelioma [22]; however, gene fusions do not appear to be a major mechanism for deregulation of ELF5, in contrast to other ETS factors, such as *TMPRSS2-ERG/ETV1* fusions in prostate cancer [23].

The breast is the most well-studied context for the role of ELF5 in cancer, with microarrays showing increased expression in basal-like subtypes and decreased expression in luminal A/B and Erb-b2 receptor tyrosine kinase 2 (HER2)-overexpressing subtypes [24, 25], suggesting subtype-specific effects. Transient ELF5 expression in cell line models reduced proliferation, invasion, ER -driven transcription and epithelial–mesenchymal transition [25, 26]. However, sustained increased ELF5 expression in some contexts is associated with disease progression, such as in endocrine-resistant breast cancers, reliant on elevated ELF5 for growth in cell line models, and the basal-like subtype of breast cancer [25]. This illustrates the complexity and contextual dependence of transcriptional regulation.

It is becoming increasingly recognized that almost all multiexon genes undergo alternative transcription (such as alternative transcription start or termination sites) and/or alternative exon splicing, increasing diversity of protein structure and function [27]. Alternative transcription events are also commonly deregulated in cancer, contributing to tumor initiation and progression but also providing potential cancer-specific therapeutic targets. Importantly, different isoforms produced by the same gene may have very different functions. One striking example is vascular endothelial growth factor, which produces both proangiogenic and antiangiogenic isoforms [28]. Early studies described tissue-specific differences in *ELF5* transcript isoform expression [6], but recent studies have not distinguished between isoforms or have used a single isoform for overexpression studies.

This study represents the first comprehensive analysis of *ELF5* expression at the isoform level, using RNA-sequencing (RNA-seq) data from The Cancer Genome Atlas (TCGA) for 6757 normal tissue and cancer samples. The functional effects of ELF5 isoform expression in breast cancer were also investigated using inducible cell line models and a 116-gene quantitative polymerase chain reaction (qPCR) panel, leading to unique insights into the transcriptional functions of ELF5 and in particular the role of the PNT domain.

## Methods

### RNA-sequencing analysis

RNA-Seq version 2 data for initial primary tumors and solid tissue normal samples (where *n* ≥ 3) were downloaded from TCGA data portal (https://tcga-data.nci.nih.gov/tcga/) [29–43], with institutional human research ethics committee exemption. Samples with available RNA-Seq version 2 data (August 2013 for breast and April 2014 for all other cancer types) were included. The RNA-Seq version 2 TCGA pipeline for preprocessing of publicly available data used MapSplice [44] for alignment and RSEM [45] for quantitation. Non-normalized gene and isoform data were downloaded from TCGA as RSEM expected (“raw”) counts, unadjusted for transcript length, and scaled estimates, adjusted for transcript length. Scaled estimates were multiplied by 10^6^ to obtain transcripts per million (TPM) values. Normalized gene and isoform data were downloaded from TCGA as quantile normalized RSEM expected counts (unadjusted for transcript length), with the upper quartile set at 1000 for gene data and 300 for isoform data.

A summary of all TCGA samples used in the analysis is shown in Table 1. For breast cancer samples, PAM50 (Predication Analysis of Microarrays 50-gene classifier) status was used to generate a subtyped cohort of 515 patients and 59 matched normal samples [29, 46]. Six additional normal samples, matching to tumors in the initial cohort, were included in differential expression analyses.

**Table 1:** Summary of all TCGA RNA-sequencing samples used in analysis

Tissue	Cancer type	TCGA acronym	Normal samples^a^	Cancer samples
Bladder	Bladder urothelial carcinoma	BLCA	19	241
Breast	Breast invasive carcinoma	BRCA	59^b^	515
	Luminal A			229
	Luminal B			126
	HER2			57
	Basal-like			96
	Normal-like			7
Cervix	Cervical squamous cell carcinoma and endocervical adenocarcinoma	CESC	3	185
Colon	Colon adenocarcinoma	COAD	41	261
Head/neck (including mouth and throat)	Head and neck squamous cell carcinoma	HNSC	43	497
Kidney	Chromophobe	KICH	25	66
	Clear cell carcinoma	KIRC	72	518
	Papillary cell carcinoma	KIRP	30	172
Liver	Hepatocellular carcinoma	LIHC	50	191
Lung	Lung adenocarcinoma	LUAD	58	488
	Lung squamous cell carcinoma	LUSC	50	490
Pancreas	Pancreatic adenocarcinoma	PAAD	3	85
Prostate	Prostate adenocarcinoma	PRAD	50	297
Rectum	Rectum adenocarcinoma	READ	9	91
Thyroid	Thyroid carcinoma	THCA	59	498
Uterus	Uterine corpus endometrial carcinoma	UCEC	24	158
	Uterine carcinosarcoma	UCS	NA^c^	57
Adrenal gland	Adrenocortical carcinoma	ACC	NA	79
Hematological	Diffuse large B-cell lymphoma	DLBC	NA	28
	Acute myeloid leukemia	LAML		173
Brain	Glioblastoma multiforme	GBM	NA	156
	Lower grade glioma	LGG		463
Ovary	Ovarian serous cystadenocarcinoma	OV	NA	262
Skin	Cutaneous melanoma	SKCM	NA	82
Bone/connective tissue/soft tissue	Sarcoma	SARC	NA	103

*TCGA* The Cancer Genome Atlas

^a^Normal samples included where *n* ≥ 3

^b^65 samples included in differential expression analysis

^c^Uterine corpus endometrioid carcinoma normal samples used as normal uterine samples for differential expression analysis

Limma voom [47] was used for differential expression analysis of gene-level RNA-seq data, with inputs as non-normalized gene data (RSEM expected counts). Filtering was applied to remove genes with low expression, keeping genes with counts >1 in at least *n* samples (where *n* = number of samples in smallest group of replicates). The trimmed mean of M-values normalization method [48] was applied, followed by differential expression analysis using Limma voom. All fold change (FC) and false discovery rate (FDR) values reported were generated by Limma voom analyses. Venn diagrams were created using online software (http://bioinformatics.psb.ugent.be/webtools/Venn/), and clustered heat maps were created using the R package gplots [49]. As a comparison, differential expression analysis was also carried out using edgeR [50–54] (see Additional file 1: Methods).

### Stable cell line generation

*ELF5* isoforms 1, 2, and 3 were tagged with C-terminal V5 (and short linker sequence), cloned into the pHUSH-ProEx vector [55], and used as a retrovirus. T47D-EcoR and MDA-MB-231-EcoR cells stably expressing ecotropic receptor were infected with pHUSH-*ELF5* retrovirus and selected using puromycin. To generate clonal cell lines, stable cell line pools were plated at low density in 96-well plates.

### Cell lines and treatments

All cell lines were obtained from the American Type Culture Collection (Manassas, VA, USA) and were maintained in RPMI medium supplemented with insulin and 10 % tetracycline-free fetal bovine serum (Clontech Laboratories, Mountain View, CA, USA). Puromycin was added at a concentration of 1 μg/ml. Doxycycline (Dox) was added at a concentration of 0.1 μg/ml daily to induce protein expression.

### Cell number assay

Cell number was quantified using a spectrophotometric assay. Cells were incubated with 16 % trichloroacetic acid and stained with 10 % Diff-Quik II solution (Lab Aids, Narrabeen, Australia). 10 % acetic acid was added to dried plates, and 100 μl of solution from each well was added to a 96-well plate, which was read at 595 nm. Absorbance readings were transformed to natural logarithms, and values from three wells (single experiment) were averaged for each time point. The minus Dox and plus Dox slopes for each cell line were compared using linear regression analysis.

### Western blot analysis

Protein was prepared in NuPAGE Sample Buffer and Reducing Agent (Life Technologies, Carlsbad, CA, USA) using 10 μg (estrogen-related blots), 65 μg (V5 blot, T47D-ELF5-isoform 2-V5) or 25 μg (V5 blots, all other lines) per lane. Samples were separated on precast 15-well 4–12 % Bis-Tris (estrogen-related blots) or 10-well 10 % Bis-Tris (V5 blots) polyacrylamide gels (Life Technologies), transferred to polyvinylidene fluoride membrane, blocked in 5 % skim milk, and incubated overnight at 4 °C in primary antibody. Secondary horseradish peroxidase–conjugated antibody was added 1:2000 in 5 % skim milk (anti-mouse, NA931V, anti-rabbit, NA934V; GE Healthcare Life Sciences, Little Chalfont, UK). Proteins were detected using enhanced chemiluminescence solution (Western Lightning Plus; PerkinElmer, Waltham, MA, USA) and x-ray film (Fujifilm, Tokyo, Japan). Primary antibodies used were anti-V5 (sc-58052, 1:500–1:1000; Santa Cruz Biotechnology, Santa Cruz, CA, USA), anti-transducin-like enhancer of split 1 (anti-TLE1) (ab183742, 1:1000; Abcam, Cambridge, UK), anti-ERα (sc-8005, 1:1000; Santa Cruz Biotechnology), anti-Forkhead box A1 (anti-FOXA1) (sc-101058, 1:1000, Santa Cruz Biotechnology), and anti-β-actin (AC-15, 1:20,000; Sigma-Aldrich, St. Louis, MO, USA).

### Transient retroviral infection

*ELF5* isoform 3 was tagged with C-terminal hemagglutinin (HA), cloned into the pQCXIH vector (Clontech) and used as a retrovirus. MDA-MB-231-EcoR-pHUSH-ELF5-isoform 2-V5 Clone 7 cells were infected with *ELF5*-isoform 3-HA/empty vector retrovirus diluted 1:4. No pQCXIH selection pressure was applied.

### Immunofluorescence

Cells were infected with pQCXIH retrovirus in eight-well Lab-Tek II chamber slides (Thermo Scientific, Waltham, MA, USA) and allowed to recover for 24 h. Dox /vehicle treatment (lasting 24 h) was then commenced. Cells were fixed with 4 % paraformaldehyde diluted in PHEM buffer (60 mM piperazine-*N*,*N*′-bis(2-ethanesulfonic acid) (PIPES), 25 mM 4-(2-hydroxyethyl)-1-piperazineethanesulfonic acid (HEPES), 1 mM ethylene glycol tetraacetic acid (EGTA), 2 mM MgCl_2_, pH 6.9), permeabilized with 0.5 % Triton X-100, blocked with 10 % donkey serum/PHEM solution, and incubated overnight at 4 °C in primary antibody. Secondary antibodies were added at 1:200, and coverslips were applied using Duolink In Situ Mounting Medium with 4′,6-diamidino-2-phenylindole (DAPI) (Olink Bioscience, Uppsala, Sweden). Imaging was performed on a Leica DM5500 microscope (Leica Microsystems, Wetzlar, Germany). Antibodies (in 10 % donkey serum/PHEM solution): anti-V5 (sc-58052, 1:200; Santa Cruz Biotechnology), anti-HA (3724, 1:800; Cell Signaling Technology, Danvers, MA, USA), and donkey anti-mouse Alexa Fluor 647 and donkey anti-rabbit Alexa Fluor 555 conjugates (1:200; Molecular Probes/Thermo Fisher Scientific, Eugene, OR, USA).

### Quantitative PCR

RNA was extracted using the RNeasy Mini Kit with DNase treatment (Qiagen, Valencia, CA, USA) and quantified using the NanoDrop spectrophotometer (NanoDrop Products, Wilmington, DE, USA). Complementary DNA (cDNA) was made using the High-Capacity cDNA Reverse Transcription Kit (Life Technologies) with ribonuclease inhibitor (Promega, Madison, WI, USA). All qPCRs were run on an ABI 7900 qPCR machine (Applied Biosystems, Foster City, CA, USA), using standard TaqMan cycling conditions or Roche Universal Probe Library (UPL) protocol with two or three technical replicates per sample (see also Additional file 1).

For the clonal cell line time-course qPCR (Fig. 6f), 0.5 μg of RNA per 20 μl of cDNA reaction and ELF5 (Hs01063022_m1) and glyceraldehyde 3-phosphate dehydrogenase (4236317E) assays were used. For the 116-gene panel, cell lines were treated for 48 h with Dox or vehicle. cDNA reactions were scaled to 100 μl and 2.5 μg RNA. Roche UPL assays were designed using the online Roche ProbeFinder software. All assays are detailed in Additional file 2.

Results were analyzed using SDS 2.4 (Life Technologies) and qbase + software (Biogazelle, Gent, Belgium) [56]. Paired *t* tests were used to calculate *p* values, comparing -Dox and + Dox samples (three or four pairs per cell line group). Correction for multiple comparisons was performed using the Benjamini-Hochberg procedure, setting the FDR at 0.10 [57].

## Results

### *ELF5* isoforms are differentially expressed in normal tissues

**Fig. 1:**
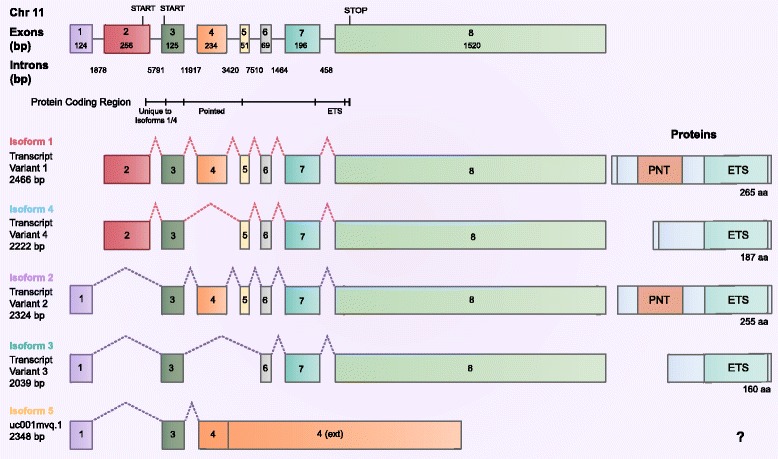
ELF5 isoforms are produced by alternative promoter use and splicing. RefSeq and GENCODE transcripts with protein products are shown. *ETS* E26 DNA-binding domain, *PNT* Pointed domain, *bp* base pairs, *aa* amino acids, *ext* extended

There are four *ELF5* transcript variants in the National Center for Biotechnology Information RefSeq database [58], predicted to produce four unique proteins (Fig. 1). The two full-length transcripts (isoforms 1 and 2) use alternative promoters, resulting in unique first exons and proteins that differ by only ten N-terminal amino acids. Two additional transcripts (isoforms 3 and 4) are produced by splicing of exons 4 (±5) from each of the full-length transcripts, producing proteins that lack the PNT domain but retain the ETS domain. An additional transcript (isoform 5), described by the GENCODE Consortium [59], is a variant of isoform 2 terminating at an extended exon 4. This type of intronic extension (“bleeding exon”) is often associated with incompletely processed transcripts [60], and it is unclear whether this transcript produces a protein product (which would lack the ETS domain).

RNA-seq data from TCGA were analyzed to quantify and compare *ELF5* isoforms in normal and cancer tissues [29–43]. A summary of all TCGA samples analyzed is shown in Table 1. TCGA preprocessed data include *ELF5* isoforms 1, 2, and 3 as annotated by RefSeq, as well as isoform 5. Due to the reference annotation used by TCGA, there are no data for *ELF5* isoform 4. The transcripts and protein products are summarized in Fig. 1, and a cross-database comparison is shown in Additional file 1: Figure S1.

**Fig. 2:**
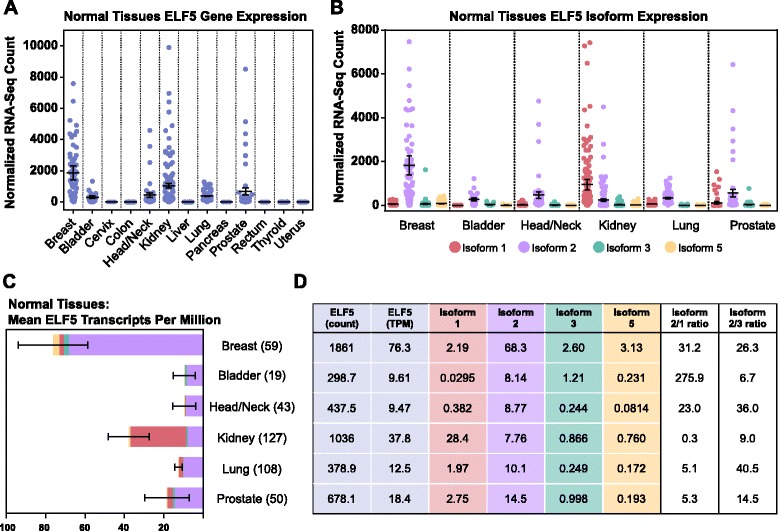
E74-like factor 5 (*ELF5*) isoforms are differentially expressed in normal tissues. Plotted values are for individual RNA-sequencing samples from The Cancer Genome Atlas, and error bars represent the mean with 95 % confidence interval. **a** *ELF5* gene expression in 13 normal tissues (quantile normalized counts). **b** *ELF5* isoform expression in selected normal tissues (quantile normalized counts). **c** Mean *ELF5* levels (transcripts per million, TPM) in normal tissues. Relative isoform contributions are shown within each bar. Numbers in parentheses indicate samples per group. **d** Mean *ELF5* gene and isoform expression in normal tissues. All values are TPM, except for column 1, which is the quantile-normalized count. Isoform ratios in the final two columns were calculated using mean TPM values

*ELF5* expression was highest in epithelial tissues such as the breast, kidney, lung, prostate, and bladder (Fig. 2a). The breast was one of the highest *ELF5*-expressing tissues in the body. Isoform 1 and 2 expression was highly tissue-specific (Fig. 2b), indicating alternative promoter use in different tissues.

Data in Fig. 2a and b were quantile-normalized by the TCGA pipeline, allowing comparison of abundance of a particular transcript (such as total *ELF5*) between samples. However, longer transcripts will generate more sequencing reads, making quantitative comparison of transcripts of different lengths problematic. To overcome this, the proportional measure TPM may be used. TPM is an example of a within-sample normalization method, and it should be noted that values are not technically comparable between samples, particularly when the composition of the total mRNA pool may be quite different (for example, when comparing different tissues). For this reason, data are shown for both quantile-normalized (between-samples–normalized) (Fig. 2b) and TPM-normalized (within-sample–normalized) (Additional file 1: Figure S2b). As the lengths of *ELF5* transcripts are not widely different, ranging from 2039 to 2466 base pairs, the data plots are in fact similar.

Since TPM is a proportional measure, the relative abundances of transcripts of different lengths within samples can be compared. The mean TPM values for *ELF5* isoforms are shown in Fig. 2c and d. Breast, bladder, head/neck, lung, and prostate all expressed isoform 2 as their main transcript (median percentage 82.1–95.2 %) (Additional file 1: Figure S2a), while the kidney expressed mainly isoform 1 (median 91.8 %). All tissues examined expressed, on average, more full-length isoform 2 than the shorter isoform 3.

### *ELF5* expression is significantly altered in cancer

**Fig. 3:**
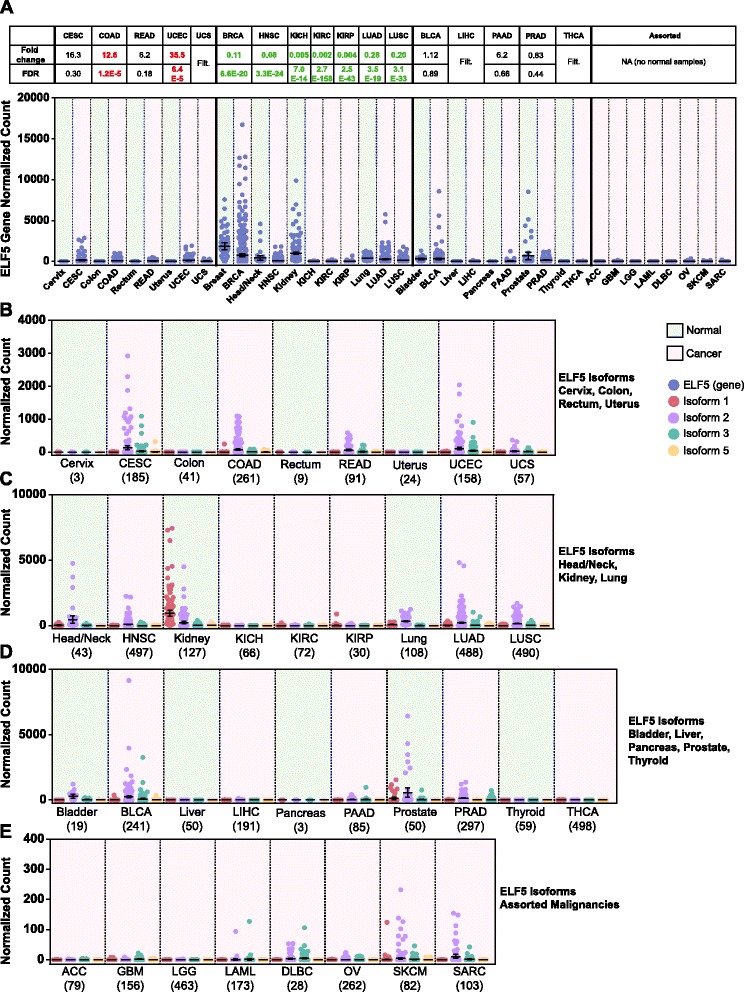
E74-like factor 5 (*ELF5*) expression is significantly altered in cancer. The Cancer Genome Atlas (TCGA) RNA-sequencing (RNA-seq) data for 25 cancer types (*pink background*) are shown, with normal tissue comparisons (*green background*) where available. Plotted values are for individual TCGA RNA-seq samples, and error bars represent the mean with 95 % confidence interval. TCGA cancer acronyms are used (see Table 1). **a** *ELF5* gene expression (normalized counts) for 25 cancers with normal tissue comparisons where available. Fold changes and false discovery rates (FDRs) from Limma voom analysis are shown, with *green* values in bold indicating significant downregulation and *red* values in bold significant upregulation compared with normal (FDR < 0.05). *Filt*. indicates gene filtered from Limma voom analysis due to low expression. **b** *ELF5* isoform expression in normal and cancer samples (with *ELF5* gene upregulation in cancer). **c** *ELF5* isoform expression in normal and cancer samples (with *ELF5* gene downregulation in cancer). **d** *ELF5* isoform expression in normal and cancer samples (unchanged or filtered *ELF5*)*.* **e** *ELF5* isoform expression in cancer samples without available normal samples (normal samples ≤ 2). Note smaller scale on *y*-axis

In malignancy, *ELF5* expression was significantly altered compared with normal tissues, as shown by Limma voom differential gene expression analysis (Fig. 3a). In the cervix, colon, rectum, and uterus, cancer was associated with an increase in *ELF5* level, driven mainly by an increase in isoform 2 and, to a lesser extent, isoform 3 (Fig. 3b). Conversely, there was almost complete suppression of *ELF5* expression in three kidney carcinoma subtypes. *ELF5* expression was also significantly decreased in head and neck, lung, and prostate cancer (Fig. 3c). In both lung carcinoma subtypes, there was a large variation in *ELF5* levels, suggesting possible molecular subtype-specific expression patterns, similar to the breast. *ELF5* expression was largely unchanged (or filtered from analysis due to low expression) in the tissues shown in Fig. 3d. The cancer types shown in Fig. 3e exhibited very low levels of *ELF5* expression but had no normal tissue samples available for comparison. Analysis of additional RNA-seq normal tissue datasets (Genotype-Tissue Expression Project and Illumina Human BodyMap) confirmed that the normal adrenal gland, brain, leukocytes/whole blood, lymph node, ovary, and skeletal muscle all had very low or absent *ELF5* expression (Additional file 1: Figure S3a and b). Skin was the only exception from this group of tissues demonstrating moderate *ELF5* expression consistent with previous studies of differentiated keratinocytes [6].

Differential expression analysis was also carried out using edgeR. Overall, the results from Limma voom and edgeR were similar. The edgeR FC and FDR values are presented in Additional file 1: Figure S4a for comparison.

### *ELF5* expression is altered in breast cancer in a subtype-specific manner

**Fig. 4:**
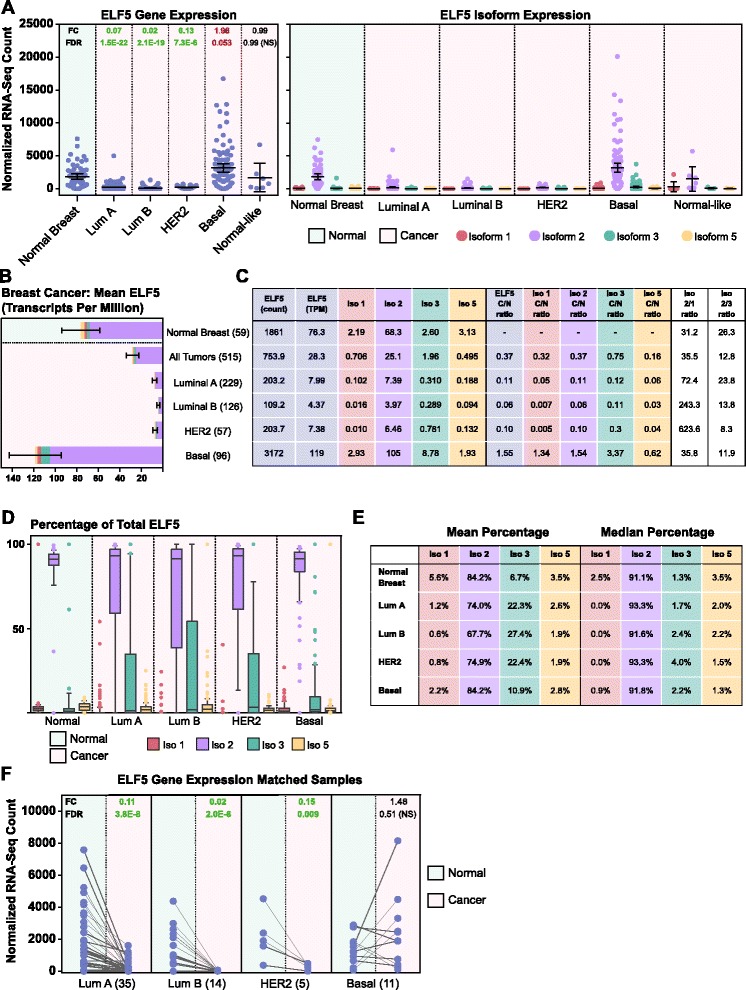
E74-like factor 5 (*ELF5*) expression is altered in breast cancer in a subtype-specific manner. **a** *ELF5* gene (*left*) and isoform (*right*) expression (quantile-normalized counts) for normal breast and breast cancer subtypes. Plotted values are for The Cancer Genome Atlas RNA-sequencing (RNA-seq) samples, and error bars represent the mean with 95 % confidence interval. Fold change (FC) and false discovery rate (FDR) from Limma voom analysis are shown for *ELF5* gene data, with *green* values in bold indicating a significant downregulation and *red* values in bold a significant upregulation compared with normal (FDR < 0.05). Nonbold *green* or *red* values indicate FDR of 0.05–0.10. **b** Mean *ELF5* levels in transcripts per million (TPM) in normal breast and breast cancer, excluding normal-like, with 95 % confidence interval. Relative isoform contributions shown within each bar. Numbers in parentheses indicate samples per group. **c** Mean *ELF5* expression values at the gene and isoform levels (columns 1–6), isoform fold changes in cancer compared with normal (columns 7–11), and isoform ratios (columns 12 and 13). All values are TPM, except for column 1, which is the quantile-normalized count. Ratios were calculated using mean TPM values. **d** Box-and-whisker plot representing isoform percentage of total *ELF5* in normal breast and cancer. *Box* 25–75th percentile, *horizontal line* median, *error bars* 10th–90th percentile, *circles* outliers. **e** Mean (left) and median (right) isoform percentage values for normal breast and cancer. **f** *ELF5* levels (quantile-normalized count) for patients with matched normal and cancer samples, categorized according to tumor molecular subtype. Six extra matched normal samples were included, for a total of 65 pairs. Plotted values represent individual samples, with samples from the same patient connected with a line. FC and FDR from paired Limma voom analysis are shown, with *green* values indicating a significant downregulation compared with normal (FDR < 0.05). Numbers in parentheses indicate sample pairs per group

Comprehensive analysis of RNA-seq incorporating molecular subtype was undertaken for 515 breast cancer patients. In the luminal A, luminal B, and HER2 subtypes, *ELF5* was significantly downregulated (fold change 0.02–0.13 compared to normal), while in the basal subtype there was a strong trend for increased *ELF5* expression (1.96-fold compared with normal, FDR 0.053 in Limma voom analysis, 1.99-fold compared with normal, FDR 0.0008 in edgeR analysis) (Fig. 4a and Additional file 1: Figure S4b). There was no clear relationship between *ELF5* expression and American Joint Committee on Cancer stage (Additional file 1: Figure S5).

This analysis was extended to the isoform level by examining the contribution to total *ELF5* (based on mean TPM) for each isoform (Fig. 4b). Normal-like samples were excluded due to low sample numbers. The main isoform expressed in all breast cancer subtypes was isoform 2. In the luminal A, luminal B, and HER2 subtypes, all *ELF5* isoforms were decreased in cancer compared with normal (Fig. 4c). Conversely, in the basal subtype, three of four isoforms were upregulated, with isoform 3 having a relatively larger fold change.

The percentage contributions of each isoform to total *ELF5* were also analyzed (Fig. 4d and e). The normal breast showed a tight range of expression, while in cancer, particularly for isoforms 2 and 3, this was broadened (Fig. 4d). The high variability in isoform 3 percentage values in the cancer samples led to an increased mean percentage in all subtypes. Median values demonstrated a smaller, although still increased, isoform 3 percentage in cancer, while the median isoform 2 percentage remained fairly constant across normal and cancer samples.

Within this cohort, 65 patients had matched tumor and normal samples that could be directly compared (Fig. 4f and Additional file 1: Figure S4c). The luminal A, luminal B, and HER2 groups showed a highly significant decrease in *ELF5* level in both the Limma and edgeR analyses. In the basal subgroup, there was an upward but variable trend.

### Expression of other ETS family members is also altered in breast cancer, with the basal subtype having a distinct ETS expression profile

The same cohort of patients was used to examine expression of other members of the ETS transcription factor family. RNA-seq data showed that a large number of ETS factors were expressed in the normal breast. Average TPM values (which take into account transcript length) for ETS factors in the normal breast ranged from 0.02 to 117.7. Several ETS factors had very low expression (<2 TPM), including *FEV*, *SPIC*, *ETV2*, *ETV3L*, and *SPIB*. The most highly expressed ETS factors in the normal breast were *EHF*, *ELF3*, *SPDEF*, and *ELF5* (Additional file 1: Figure S6).

ETS factor expression was significantly altered in breast cancer, as shown by Limma voom differential expression analysis. In the first (unpaired) analysis, samples from each molecular subtype, excluding normal-like, were compared with the common set of 65 normal breast samples, allowing analysis of larger sample sets. In the second (paired) analysis, normal and subtyped tumor samples from the same patient were compared, allowing for more rigorously matched comparisons but limited by smaller sample numbers. ETS factors with low expression (three to five per subtype) were filtered from the analysis.

**Fig. 5:**
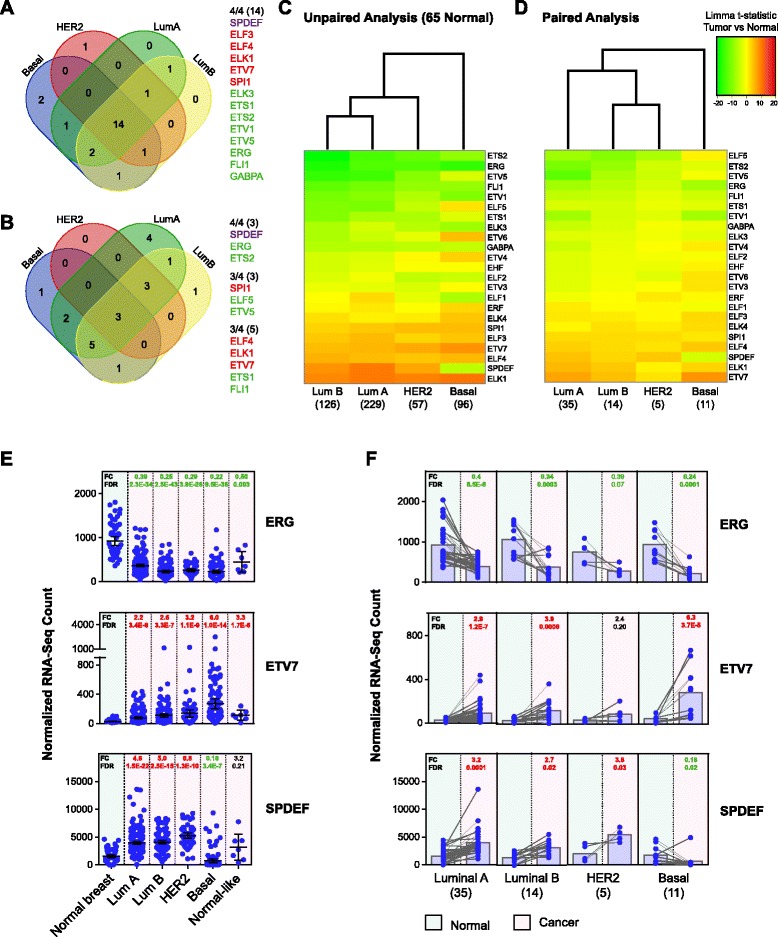
Expression of other E26 transforming sequence (ETS) family members is also altered in breast cancer, with the basal subtype having a distinct ETS expression profile. The Cancer Genome Atlas RNA-sequencing (RNA-Seq) Limma voom differential expression analysis data for ETS family members. **a** Venn diagram showing number of ETS family members significantly altered in breast cancer subtypes compared with normal (false discovery rate [FDR] < 0.05). All subtypes were compared with a common set of 65 normal samples (unpaired analysis). Genes altered in all four subtypes are listed (*red* = upregulation, *green* = downregulation, *purple* = differentially regulated in basal subtype compared with other subtypes). **b** Venn diagram showing number of ETS family members significantly altered in breast cancer subtypes compared with normal (FDR < 0.05), using paired normal and tumor samples from the same patient. Genes altered in at least three of four subtypes are listed, with color-coding as above. **c** Clustered heat map of ETS factor Limma voom *t* statistic, comparing tumor samples to the common set of 65 normal samples. Legend is shown next to (**d**). Rows are sorted by luminal B values (smallest to largest), and columns are sorted according to clustering. Numbers in parentheses are samples per group. **d** Clustered heat map of Limma voom *t* statistic, comparing paired normal and tumor samples, with sorting as above. Numbers in parentheses are sample pairs per group. **e** Expression of *ERG*, *ETV7*, and *SPDEF* for normal breast (*green* background) and breast cancer subtypes (*pink* background). Plotted values are for individual samples (normalized counts), and error bars represent the mean with 95 % confidence interval. Fold change (FC) and FDR from unpaired Limma voom differential expression analysis are shown, with *green* indicating a significant downregulation and *red* a significant upregulation compared with normal (FDR < 0.05). **f** *ERG*, *ETV7*, and *SPDEF* levels for a 65 patients with matched normal and cancer samples. FC and FDR from paired Limma voom differential expression analysis are shown, with color-coding as above (FDR < 0.05). Numbers in parentheses are sample pairs per group

Of the 25 ETS factors included in the unpaired analysis, 24 were significantly altered in at least 1 subtype, with 14 common to all subtypes (Fig. 5a). Within these, 13 were altered in the same direction (5 up and 8 down in the tumor compared with normal), while *SPDEF* was oppositely regulated in basal compared with other subtypes. In the paired analysis, 21 ETS factors were significantly altered in at least 1 subtype, with 3 ETS factors common to all subtypes (*SPDEF*, *ERG*, and *ETS2*) and an additional 8 common to 3 of 4 subtypes (Fig. 5b). *ELF5* was the most downregulated ETS family member by fold change in the luminal A, luminal B, and HER2 subtypes in both unpaired and paired analyses.

Compared with other subtypes, the basal group showed a number of unique ETS factor expression changes. To further explore this, the Limma *t* statistics for all ETS family members (tumor compared with normal) were plotted on a clustered heat map (Fig. 5c, unpaired, and Fig. 5d, paired). The basal subtype showed a distinct expression profile and clustered separately from the other subtypes in both paired and unpaired analyses, highlighting the potential for the ETS transcription factor family to exert a unique transcriptional influence in this subtype. Similar results were obtained with unpaired and paired edgeR analyses (Additional file 1: Figure S7).

Several ETS family members with significant changes in expression were selected to visualize the results of the breast cancer differential expression analyses. The normalized counts for *ERG* (downregulated), *ETV7* (upregulated), and *SPDEF* (differentially regulated) are shown in Fig. 5e. Direct comparison of matched normal and tumor samples is shown in Fig. 5f. Interestingly, *SPDEF* showed the inverse expression pattern of *ELF5*. The normalized counts for the entire ETS factor family, with the results of the Limma voom and edgeR differential expression analysis, are shown in Additional file 1: Figure S8.

### Alterations in cell line *ELF5* isoform levels result in a similar phenotype, characterized by decreased cell number, decreased estrogen-related proteins, and nuclear localization

TCGA data showed an increased diversity of *ELF5* isoform expression in cancer compared with the normal breast; therefore, the expression levels and effects of ELF5 isoform expression were examined in vitro to determine if this was of functional consequence.

ELF5 expression in a panel of breast cancer cell lines was analyzed by qPCR and Western blotting (Additional file 1: Figure S9a and d). Three cell lines (T47D, BT474, and HCC1187) expressed high levels of ELF5 protein (Additional file 1: Figure S9d), with the size of the main band consistent with isoform 2. A possible band representing isoform 3 was seen in the HCC1187 cell line; however, interpretation was difficult due to high background.

Clonal cell lines were constructed with a Dox-inducible expression vector containing a single ELF5 isoform, tagged with C-terminal V5. The luminal cell line T47D (ER+/PR+/HER2−) was chosen to examine the effect of isoforms in the context of relatively high endogenous ELF5 expression, testing the hypothesis that isoforms lacking the PNT domain might exert a dominant-negative effect on full-length isoform function. A second claudin-low cell line, MDA-MB-231 (ER−/PR−/HER2−), was chosen as it expresses no endogenous ELF5, allowing the effects of each isoform to be determined in the absence of potential competitive isoform interactions.

**Fig. 6:**
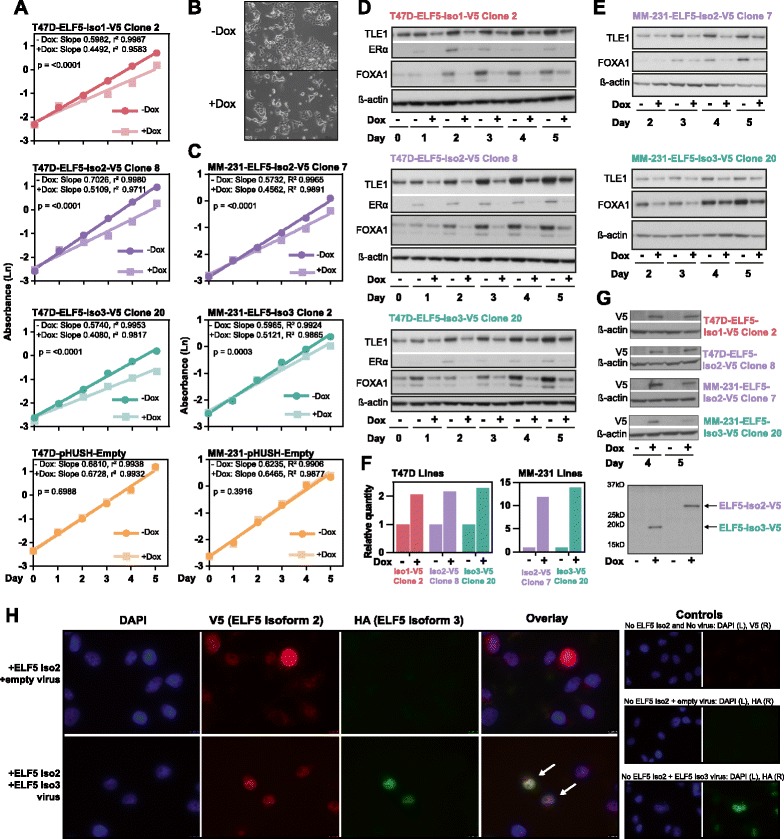
Alterations in cell line E74-like factor 5 (ELF5) isoform levels result in a similar phenotype, characterized by decreased cell number, decreased estrogen-related proteins, and nuclear localization. **a** and **c** Time course of T47D (**a**) and MDA-MB-231 (**c**) pHUSH clonal cell line growth with and without doxycycline (Dox) over 5 days. Graphs show the natural logarithm (Ln) of spectrophotometric assay absorbance value (*y*-axis) plotted against day (*x*-axis). *p* values compare minus Dox and plus Dox slopes for each cell line. One experiment is shown. **b** Representative light microscopic images of T47D cells with and without doxycycline, taken at day 4. **d** Western blots for estrogen-related proteins from T47D time courses, days 0–5. **e** Western blots for estrogen-related proteins from MDA-MB-231 time courses, days 2–5. **f** Quantitative polymerase chain reaction for *ELF5* (day 5 time course samples) minus and plus Dox. **g** Western blots for V5 at days 4 and 5 minus and plus Dox, 65 μg per lane (T47D-ELF5-isoform 2-V5 line) or 25 μg per lane (all others). *Bottom panel* shows representative samples from MDA-MB-231 cell lines, demonstrating size difference between isoforms 2 and 3. **h** Immunofluorescent images of MDA-MB-231-ELF5-isoform 2-V5 clone 7 cells. *Blue* = nuclei (4′,6-diamidino-2-phenylindole [DAPI]), *red* = V5, *green* = hemagglutinin (HA). *Arrows* mark cells with double-isoform 2 and 3 expression

Over a 5-day time course, induced expression of isoforms 1, 2, and 3 all resulted in a significantly decreased growth rate in T47D cells, with no change in the empty vector control (Fig. 6a). Representative light microscopic images for T47D lines (Fig. 6b) demonstrate decreased cell number and increased detached cells (additional images shown in Additional file 1: Figure S9e and f). A similar but less pronounced decrease in growth rate was also seen with induction of isoform 2 and isoform 3 in the MDA-MB-231 lines (Fig. 6c). It has previously been shown that the mechanisms underlying this phenotype for ELF5 isoform 2 include G_1_ arrest, increased apoptosis, and reduced adhesion proteins [25].

In the T47D lines, each isoform caused a decrease in ERα protein and pioneer factors FOXA1 and TLE1, required for ER–chromatin interactions [61, 62] (Fig. 6d). The effects on FOXA1 and TLE1 were also seen in the MDA-MB-231 lines, in the absence of detectable ERα (Fig. 6e). Dox -inducible *ELF5* mRNA expression was shown by qPCR (day 5) (Fig. 6f). V5 antibody Western blot analysis confirmed ELF5-V5 protein expression and also illustrated the size difference between isoforms 2 and 3 (Fig. 6g).

Immunofluorescence was performed to determine the subcellular location of ELF5 isoforms when expressed in isolation and when coexpressed. MDA-MB-231 cells with Dox -inducible ELF5-isoform 2-V5 expression were used, with transient retroviral infection of an ELF5-isoform 3-HA vector. This allowed manipulation of isoform 2 and isoform 3 levels within the same cell. Figure 6h (*top row*) shows MDA-MB-231-ELF5-isoform 2-V5 cells treated with Dox to induce expression, as well as transient infection of a control pQCXIH vector. There was strong nuclear V5 staining and no HA staining. In row 2, cells were treated with Dox to induce ELF5-isoform 2-V5 and also infected with isoform 3-HA. Both isoform 2 (V5) and isoform 3 (HA) localized to the nucleus, and there was no cytoplasmic redistribution seen in the cells that expressed both isoform 2 and isoform 3 (indicated by *arrows*), an effect that has been reported previously for ETS1 isoforms [63].

### ELF5 isoforms have a similar transcriptional effect in T47D and MDA-MB-231 cell lines

A panel of 116 genes was examined by qPCR to compare the transcriptional effects of *ELF5* isoforms. Previously published microarrays and ELF5/V5 chromatin immunoprecipitation with massively parallel DNA sequencing [25] were used to identify genes and pathways regulated by ELF5 isoform 2 in luminal cell lines. The assays are described in Additional file 2, with an outline of the experimental workflow shown in Additional file 1: Figure S10.

**Fig. 7:**
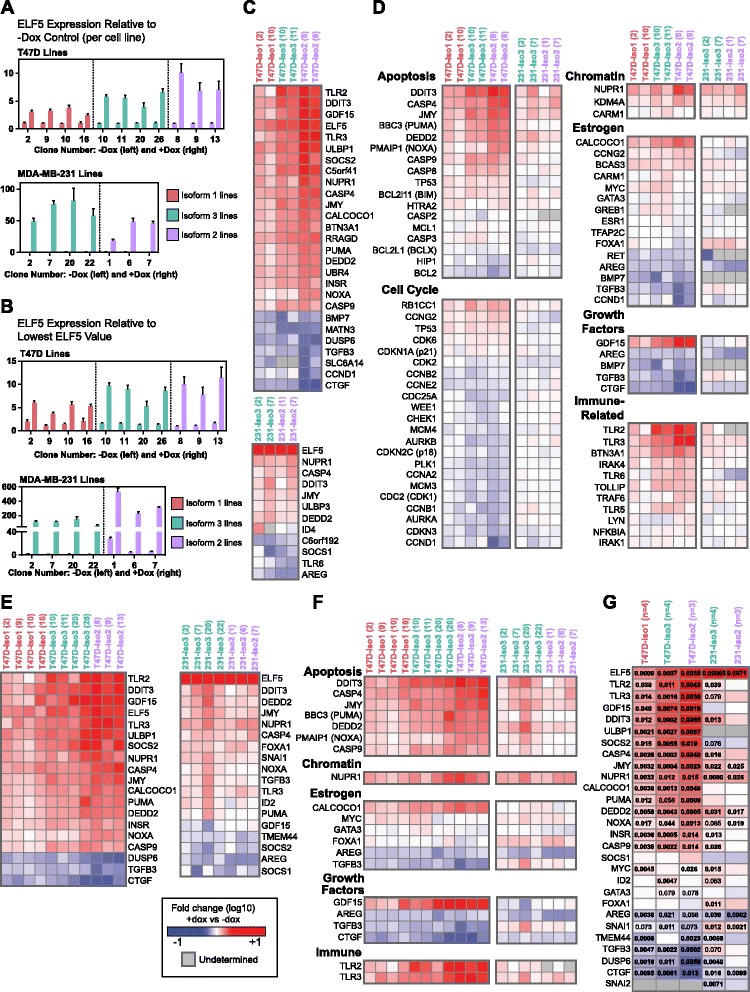
E74-like factor 5 (ELF5) isoforms have a similar transcriptional effect in T47D and MDA-MB-231 cell lines. **a** *ELF5* expression measured by quantitative polymerase chain reaction (qPCR) at 48 h for T47D clonal cell lines (*top*) and MDA-MB-231 clonal cell lines (*bottom*). Assay detects all *ELF5* isoforms. Values are the mean calibrated normalized relative quantity (CNRQ) with standard error. Results relative to the minus doxycycline (−Dox) control (set at 1) for each cell line. **b** *ELF5* expression measured by qPCR at 48 h for T47D clonal cell lines (*top*) and MDA-MB-231 clonal cell lines (*bottom*) used in the qPCR panel. Assay detects all *ELF5* isoforms. Values are the mean CNRQ with standard error. Results relative to the sample with the lowest *ELF5* value (set at 1), which is T47D-ELF5-isoform 2-V5 clone 8 (T47D lines) and MDA-MB-231-ELF5-isoform 3-V5 clone 22 (MDA-MB-231 lines). **c** Heat map showing genes (from 116-gene qPCR panel) with absolute fold change >3 (any T47D line) or >2 (any MDA-MB-231 line). Two clonal cell lines were tested per group. All heat maps use the legend shown in (**e**) and represent the log_10_ fold change (capped at −1 and +1) of the plus Dox quantity compared with the minus Dox quantity as measured by qPCR. *Gray* indicates gene was not detectable by qPCR in minus and/or plus Dox samples. **d** Functional categorization of selected genes from 116-gene qPCR panel. Some genes are represented more than once due to multiple functions. **e** Heat map showing genes (from 27-gene qPCR panel) with absolute fold change >3 (any T47D line) or >2 (any MDA-MB-231 line). Results shown for three or four clonal lines per group. **f** Functional categorization of selected genes from 27-gene qPCR panel. **g** Heat map representing the mean log_10_ fold change per group for all genes in the 27-gene panel, as well as *ELF5*. Significant *p* values are shown where false discovery rate (FDR) is <0.10. Some *p* values (nonbold) are >0.05, although FDR for these values is <0.10. Nonsignificant *p* values (FDR >0.10) are not shown

The pHUSH clonal cell lines were selected on the basis of similar qPCR levels of *ELF5* isoform induction. Figure 7a shows the *ELF5* level with Dox relative to the without Dox control for each individual cell line. To compare baseline (without Dox) variability, values were also normalized to the lowest ELF5 value (Fig. 7b). Baseline variability was minimal in the T47D lines; however, expression ranged from 1.0- to 2.3 in the MDA-MB-231 isoform 3 lines and from 4.7 (clone 6) to 28.0 (clone 1) in the isoform 2 lines. This variation is most likely due to slight “leakiness” of the pHUSH vector, leading to low-level ELF5 expression (undetectable by V5 Western blotting) in the absence of Dox.

T47D and MDA-MB-231 clonal cell lines were treated with Dox or vehicle for 48 h to induce ELF5 isoform expression. Initially, two clones per parental cell line were used. A selection of 27 genes was then repeated in 1 or 2 further clones, giving a total of 3 or 4 clonal lines (biological replicates) per parental line (Additional file 1: Table S1). The heat maps in Fig. 7 show the log_10_ FC for each gene when ELF5 isoform expression is induced (+dox) compared with baseline (−dox).

Overall, the pattern of change was fairly similar, regardless of which ELF5 isoform was expressed. The genes with the strongest absolute FC (>3 in any T47D line or >2 in any MDA-MB-231 line) showed a particularly consistent pattern of change (Fig. 7c). Expression changes were greater in the T47D than in the MDA-MB-231 cell lines.

Genes were also analyzed in functional categories (Fig. 7d). Apoptosis-related genes showed consistent changes corresponding to an increase in apoptosis, such as upregulation of apoptosis-promoting genes, including *DDIT3*, *PUMA*, *NOXA*, *TP53*, and various caspases, as well as downregulation of apoptosis-inhibiting genes such as *BCLX* and *BCL2*. The changes in cell cycle genes were weaker, although still generally consistent, with upregulation of cell cycle inhibitors such as *RB1CC1* and *TP53* and downregulation of cell cycle–promoting genes such as cyclins D1, B1, A2, and E2 and associated kinases *CDK1/2*. However, the pattern of change was not entirely congruent with inhibition of the cell cycle, with upregulation of the cyclin D–associated *CDK6* and downregulation of the cell cycle inhibitor *CDKN2C* (p18). Changes in mRNA expression for key genes associated with estrogen action, such as *ESR1*, *FOXA1*, *GATA3*, and *GREB1*, were relatively small and variable (Fig. 7d), in contrast to results at the protein level, which showed robust downregulation of ESR1 and FOXA1 with all ELF5 isoforms.

The results were substantiated using 1 or 2 further clones per parental cell line and 27 genes from the original panel (Fig. 7e and f). The average FC for each parental cell line group (consisting of three or four clonal cell lines) was calculated, and this is shown in the heat map in Fig. 7g with corresponding significant *p* values (FDR < 0.10). Although the pattern of change was generally consistent, there were some interesting differences. First, *FOXA1* expression in the T47D lines exhibited a mostly downward trend, although there were no statistically significant changes. Conversely, in the MDA-MB-231 lines, *FOXA1* mRNA increased (significant only in the isoform 2 group); again, this is in contrast to the protein results shown for the MDA-MB-231 lines in Fig. 7g. Second, there was only one case in the T47D lines (and none in the MDA-MB-231 lines) in which a gene was altered in statistically significant opposite directions by different ELF5 isoforms. This gene, *GATA3*, was upregulated by isoform 3 and downregulated by isoform 2, although the changes were relatively small. In fact, 20 of 27 genes in the T47D lines showed a statistically significant change in the same direction with each of the 3 isoforms, pointing toward the overall consistency of the transcriptional effect of ELF5 isoforms.

## Discussion

This study is the first detailed analysis of ELF5 isoform expression and function, extending previous ELF5 Northern blot analysis, immunohistochemistry, and microarray studies [5, 6, 16, 25] to the isoform level using 6757 sequenced normal and cancer samples. The kidney appears to be unique in being the only tissue examined to express isoform 1 as its dominant isoform, expanding on the initial Northern blot analysis–based descriptions of *ELF5* isoforms [6]. In breast cancer, *ELF5* alterations were subtype-specific, with the basal subtype demonstrating unique *ELF5* isoform expression changes. Despite differences in protein domains, the in vitro phenotypic and transcriptional effects of increased ELF5 isoform expression were similar. This suggests that ELF5 action is regulated in various tissues by tissue-specific alternative promoter use rather than by differences in the transcriptional activity of the isoforms.

In cancer, *ELF5* expression is frequently altered. The kidney, one of the highest *ELF5*-expressing tissues, showed a dramatic decrease in *ELF5* level in cancer. ELF5 has been characterized as a tumor suppressor in the kidney and bladder [19, 20], and this may restrict kidney carcinomas to non-*ELF5*–expressing cells of origin. In other tissues, cancer was associated with an aberrant increase in *ELF5* expression, as seen in the cervix, colon, rectum, and uterus. This may indicate an oncogenic role for ELF5 in these tissues or broader genomic deregulation, such as DNA hypomethylation, a hallmark of the cancer genome [64]. The mechanisms regulating ELF5 in different tissues and in cancer have not been widely studied; however, in the early embryo and the developing mammary gland, ELF5 regulation of lineage specification is associated with promoter methylation status [65, 66]. Increased *ELF5* promoter methylation has also been demonstrated in bladder carcinoma [19]. These studies establish DNA methylation as an important epigenetic mechanism regulating *ELF5* expression, with possible aberrant methylation in cancer.

The normal human breast expresses relatively high levels of *ELF5*, with subtype-specific alterations in cancer. High ELF5 has been shown to maintain the ER− basal phenotype, paralleling the normal developmental role of specification of the ER− alveolar lineage [25]. In all breast cancer subtypes, there was a broader distribution of *ELF5* isoform expression. Increased variability of isoform distribution (“transcriptome instability”) is a known phenomenon and is proposed as a molecular hallmark of cancer [67, 68]. A recent study identified 244 cancer-associated isoform “switches” involving consistent changes in the most abundant isoform [69]. An ELF5 isoform switch has not been identified in breast cancer, in keeping with the present study, which showed an inconsistent pattern of isoform expression variation. Although not consistently identified, this does not mean that ELF5 isoform switches do not play an important role in the subset of patients in which they occur.

Other ETS transcription factors have also been shown to be important in breast cancer. Extension of RNA-seq analysis to the entire ETS family revealed a number of cancer-associated expression changes. The ETS family as a whole has previously been studied in breast cancer at the qPCR level in mouse models [70] and human cell lines [71], although the present study is the first, to our knowledge, to include examination of the expression of the entire human ETS family in both the normal breast and subtyped breast cancer samples using RNA-seq data. The normal human breast expressed a diverse range of ETS factors. Compared with the normal breast, the basal-like subtype showed a distinct pattern of ETS factor expression changes, with several ETS factors changing in the opposite direction in basal compared with other subtypes. *ELF5* and *SPDEF* were the most striking examples of this phenomenon. *SPDEF* is also a luminal epithelial lineage-specific transcription factor in the breast and has been shown to promote the survival of ER+ breast cancer cells [72]. The inverse relationship seen between these two transcription factors in breast cancer is intriguing and may well have a parallel during normal mammary development.

Finally, the phenotypic and transcriptional effects of isoforms 1, 2, and 3 were found to be similar in inducible cell line models. This was unexpected, as the PNT domain in murine ELF5 has previously been shown to have strong transactivation activity [12]. In many proteins, SAM and/or PNT domains act as protein–protein interaction modules, an important mechanism of biological specificity for ETS factors, which often bind only weakly to DNA in the absence of binding partners or posttranslational modifications [3, 12]. The importance of the PNT domain is also shown by other ETS family members in which removal of the PNT domain significantly alters protein function. The endogenous ETS1 isoform p27, for example, lacks the PNT and transactivation domains and negatively regulates full-length ETS1 by competing for DNA-binding sites and promoting its translocation from the nucleus to the cytoplasm [63]. Although this splicing event is similar to those that occur to produce ELF5 isoforms 3 and 4, it appears that ELF5 isoform 3 can alter gene transcription in a very similar way to the full-length isoforms. In addition, there was no subcellular relocation of full-length isoform 2 seen when isoform 3 was coexpressed. Interestingly, however, while exogenous ELF5 localized to the nucleus in this study, cytoplasmic ELF5 staining is seen in some human breast cancer samples and is a predictor of outcome [73]. This indicates that endogenous ELF5 can localize to the cytoplasm and that this has functional significance in breast cancer. A potential nuclear export sequence exists in the ETS domain of ELF5 (amino acids 165–174) similar to one identified in ELF3 [74, 75]. It is possible that cytoplasmic relocation of ELF5 is mediated by the relative amounts of isoforms but that this effect is not recapitulated by exogenous expression, particularly in the context of MDA-MB-231 cells, which do not normally express ELF5 and therefore may be lacking essential protein binding partners. Given the importance of context in the function of ETS factors, it is possible that the differential effects of ELF5 isoforms may also require a stimulus (for example, growth factors) or challenge (for example, estrogen deprivation) in order to become apparent, an avenue that was not explored in this study.

## Conclusions

This study has characterized the expression pattern and functions of ELF5 at the isoform level, demonstrating significantly altered expression in cancer. Alterations in ELF5 isoform expression in cancer may drive abnormal cell fate decisions, suggesting that ELF5, like other ETS factors, may be a significant contributor to tumorigenesis. While further studies are needed to clarify the mechanisms that regulate differential ELF5 isoform expression and to fully elucidate the role of the PNT domain, understanding expression and function at the isoform level is a vital first step in realizing the potential of transcription factors such as ELF5 as prognostic markers or therapeutic targets in cancer.

## Additional files

Additional file 1:
**Methods for additional figures, extended methods for qPCR experiments, Table S1 describing all clonal cell lines, additional Figures S1–S10 including legends as described in the main text.** (PDF 11918 kb) Additional file 2:
**Excel spreadsheet with details of all qPCR assays (Roche UPL assays worksheet 1 and TaqMan assays worksheet 2).** (XLSX 25 kb)

## Abbreviations

cDNA: complementary DNA
CNRQ: Calibrated Normalized Relative Quantity
Dox: doxycycline
ELF5: E74-like factor 5
ER: estrogen receptor-α
ETS: E26 transforming sequence
FC: fold change
FDR: false discovery rate
FOXA1: Forkhead box A1
HA: hemagglutinin
HER2: Erb-b2 receptor tyrosine kinase 2
PAM50: Prediction Analysis of Microarrays 50-gene classifier
PNT: Pointed domain
PR: progesterone receptor
qPCR: quantitative polymerase chain reaction
RNA-seq: RNA sequencing
SAM: sterile alpha motif domain
TCGA: The Cancer Genome Atlas
TGF-β: transforming growth factor-β
TLE1: transducin-like enhancer of split 1
TPM: transcripts per million, a proportional measure of abundance correcting for transcript length
UPL: Roche Universal Probe Library
