# Supplementary material for: ELF5 isoform expression is tissue-specific and significantly altered in cancer
Source: Breast Cancer Res. 2016 Jan 7;18:4. doi: 10.1186/s13058-015-0666-0 (PMC4704400; doi:10.1186/s13058-015-0666-0)
Supplement: Additional file 1: — Methods for additional figures, extended methods for qPCR experiments, Table S1 describing all clonal cell lines, additional Figures S1–S10 including legends as described in the main text. (PDF 11918 kb) [file 13058_2015_666_MOESM1_ESM.pdf]

__ADDITIONAL FILE 1__

ADDITIONAL INFORMATION - METHODS

RNA-Sequencing Analysis

As a comparison to Limma voom, differential expression analysis was also carried out using edgeR [1-5], with inputs as non-normalized gene data (RSEM expected counts) rounded to the nearest integer. Filtering was applied, keeping genes with count >1 in at least *n* samples (where *n* = number of samples in smallest group of replicates). A classic edgeR approach was used for analysis of unpaired data, while a glm approach was used for paired data. Venn diagrams were generated using online software (http://bioinformatics.psb.ugent.be/webtools/Venn/) and clustered heat maps using the R package 'gplots' [6].

Additional RNA-seq datasets, including The Genotype-Tissue Expression (GTEx) dataset [7] and the Illumina Human Body Map (Additional Figure 4), were access through the EMBL-EBI Expression Atlas (www.ebi.ac.uk/gxa/experiments) using accession numbers E-MTAB-2919 and E-MTAB-513.

Plots comparing TCGA breast cancer stage and *ELF5* expression were generated using cBioPortal [8, 9].

Endpoint PCR

RNA was extracted using the RNeasy Mini Kit with DNase treatment (Qiagen). cDNA was made from 2ug RNA using the High Capacity cDNA Reverse Transcription Kit (Life Technologies) with RNasin Ribonuclease Inhibitor (Promega). PCR reactions were run for 25 cycles using the PCR Reagent System (Life Technologies) with optimized annealing temperatures and magnesium concentrations. *ELF5* Isoform 2/3 primers designed using NCBI Primer-BLAST (5' to 3'): AGCGCCTGCCTTCTCTTGCC (forward) and CCCCACATCTTTGCCAGGGCTT (reverse). Amplicons were visualized on a 1% agarose/ethidium bromide gel.

Western Blots

Breast cancer cell lines were classified according to molecular subtype [10]. Protein was prepared in NuPAGE Sample Buffer and Reducing Agent using 30ug protein (V5 blot, Additional Figure 9C) or the maximum loading volume (11.7ul lysate) capped at 100ug (ELF5 blot, Additional Figure 9D, 40ug used for control lanes). Samples were separated on pre-cast 26-well 4-12% Bis-Tris gels run in MOPS buffer (V5 blot) or MES buffer (ELF5 blot) and transferred to PVDF membrane. V5 blot was blocked in 5% skim milk and incubated overnight at $4^{o}C$ in primary antibody diluted in TBS/BSA solution. Following optimization experiments, ELF5 blot was blocked in freshly made 5% donkey serum in TBS-tween and incubated overnight at $4^{o}C$ in primary antibody also diluted in 5% donkey serum in TBS-tween. Secondary HRP-conjugated antibody was added 1:5000 in 5% skim milk (V5 blot, anti-rabbit, NA934V, GE Healthcare) or 1:5000 in 5% donkey serum in TBS-tween (ELF5 blot, anti-goat, sc-2020, Santa Cruz). Proteins were detected using enhanced chemiluminescence solution (Western Lightning Plus, Perkin Elmer) and x-ray film (Fujifilm). Primary antibodies: anti-V5 (13202, Cell Signaling Technology, 1:1000) and anti-ELF5 N-20 (sc-9645, Santa Cruz, 1:1000), anti-β-actin (AC-15, Sigma, 1:20,000). ELF5 N-20 antibody experimentally detected ELF5 Isoforms 1, 2 and 3 in overexpressing cell lines (data not shown).

Quantitative PCR

For the cell line panel (T47D, MCF7, HCC1937, HCC1187, MDA-MB-468, Supplementary Figure 9A), RNA was extracted from frozen cell pellets using the RNeasy Mini Kit (Qiagen) and quantified using the Nanodrop spectrophotometer. cDNA was made using the High Capacity cDNA Reverse Transcription Kit (Life Technologies) with RNasin Ribonuclease Inhibitor (Promega) according to the manufacturer's protocol. 2ug of RNA was used per 20uL cDNA reaction. 4.5uL cDNA (diluted 1:5 in nuclease-free water) and 5.5uL Taqman assay (diluted 1:11 in Taqman Gene Expression Mastermix, Life Technologies) were added per 10uL reaction in a 384-well plate. qPCR reactions were run for 40 cycles on the ABI7900 qPCR machine (Life Technologies) using default Taqman cycling conditions. Three technical replicates

were run for each sample, as well as standard curves using a 1:10 dilution series to determine amplification efficiency. Taqman assays used were: ELF5 (Hs00154971_m1, for Isoforms 2/3), ELF5 (Hs01063022_m1, for all variants) and GAPDH (4326317E). A custom Taqman assay was designed to detect Isoforms 1/4, using primers spanning the exon 2/3 boundary: GCCAGCTCTGAGAAGGGTTCA (forward primer), TGTGTGTCACCGAGTCCAACAT (reverse primer) and CTGTGGGAGTGAGGCAG (probe). Results were analyzed using SDS 2.4 (Life Technologies) and qbase+ software (Biogazelle) [11]. The Pfaffl method [12] was used by qbase+ to calculate relative quantities normalized to a single reference gene (GAPDH).

The clonal cell line timecourse qPCR used 0.5ug RNA per 20uL cDNA reaction and assays Hs01063022_m1 (ELF5) and 4326317E (GAPDH). Three technical replicates were run for each sample, as well as standard curves using a 1:10 dilution series. Results were analyzed using SDS 2.4 software and normalized relative quantities.

For the 116-gene qPCR panel, doxycycline-inducible cell lines were treated for 48 hours with doxycycline or vehicle prior to collection. RNA was extracted from frozen cells using the RNeasy Mini Kit with on-column DNase treatment (Qiagen) and quantified using the Nanodrop spectrophotometer. cDNA was made using the High Capacity cDNA Reverse Transcription Kit (Life Technologies) with RNasin Ribonuclease Inhibitor (Promega) according to the manufacturer’s protocol, with reactions scaled up to 100uL and 2.5ug RNA. cDNA was diluted 1:10 in nuclease-free water.

Taqman assays for the qPCR panel were run as above using 4.5uL of diluted cDNA and 5.5uL of diluted assay, with standard Taqman cycling conditions and 2 technical replicates per sample. Roche Universal Probe Library (UPL) assays were designed using the online Roche ProbeFinder software. All Roche assays were tested prior to use with a 6-point 1:10 dilution series and assays with poor amplification were not used. Each 10uL Roche qPCR reaction included 0.4uL forward primer (10uM), 0.4uL reverse primer

(10uM), 0.1uL UPL probe, 5uL LightCycler 480 Probes Master reaction mix (Roche) and 4.1uL of 1:10 diluted cDNA. Reactions were run in 384-well plates on the ABI7900 qPCR machine (Life Technologies) using the Roche UPL protocol (denature 94°C for 10 mins, cycle 94°C for 15 sec/60°C for 30 sec/72°C for 15sec (x45), cooling 40°C for 2 mins). A standard curve using a 1:10 dilution series, as well as No Template and No Reverse Transcriptase controls, were included for every Taqman and Roche assay. A complete list of all assays used, along with test and experimental amplification efficiencies, can be found in supplementary document 1.

Results were analyzed using SDS 2.4 (Life Technologies) and qbase+ software (Biogazelle) [11]. Quality control checks included exclusion of clear outliers in technical replicates, exclusion of samples with a Ct value <4-5 cycles away from a negative control and exclusion of samples amplifying with a Ct greater than 36.0-38.0 (assay-dependent). The Pfaffl method [12] was used by qbase+ to calculate Normalized Relative Quantities (NRQ), which were normalized to a single reference gene (GAPDH) with error propagation. qPCR plates were laid out so that all samples for a single assay (in each qPCR round) were run on the same plate, known as a sample maximization approach [11]. To compare the results of assays run in both rounds 1 and 2 (on different plates), inter-run calibration was performed using the qbase+ software, based on at least 3 identical samples that were run on both plates. This process calculates a calibration factor for each assay that corrects for any run-to-run differences, generating Calibrated Normalized Relative Quantity (CNRQ) values [11].

All statistical analysis of qPCR results was performed with qbase+ software using log-transformed CNRQ values. Paired *t* tests were used to calculated p-values, comparing -dox and +dox samples (3-4 pairs per cell line group). Correction for multiple comparisons was performed using the Benjamini-Hochberg procedure, setting the False Discovery Rate (proportion of significant results that are actually false positives) at 0.10 [13, 14].

REFERENCES FOR ADDITIONAL INFORMATION

1. Robinson MD, Smyth GK: **Moderated statistical tests for assessing differences in tag abundance**. *Bioinformatics* 2007, **23**(21):2881-2887.
2. Robinson MD, Smyth GK: **Small-sample estimation of negative binomial dispersion, with applications to SAGE data**. *Biostatistics* 2008, **9**(2):321-332.
3. Robinson MD, McCarthy DJ, Smyth GK: **edgeR: a Bioconductor package for differential expression analysis of digital gene expression data**. *Bioinformatics* 2010, **26**(1):139-140.
4. McCarthy DJ, Chen Y, Smyth GK: **Differential expression analysis of multifactor RNA-Seq experiments with respect to biological variation**. *Nucleic Acids Res* 2012, **40**(10):4288-4297.
5. Zhou X, Lindsay H, Robinson MD: **Robustly detecting differential expression in RNA sequencing data using observation weights**. *Nucleic Acids Res* 2014, **42**(11):e91.
6. Warnes GR, Bolker B, Bonebakker L, Gentleman R, Huber W, Liaw A, Lumley T, Maechler M, Magnusson A, Moeller S *et al*: **gplots: Various R Programming Tools for Plotting Data. R package version 2.17.0**. *http://CRANR-projectorg/package=gplots* 2015.
7. Consortium GT: **Human genomics. The Genotype-Tissue Expression (GTEx) pilot analysis: multitissue gene regulation in humans**. *Science* 2015, **348**(6235):648-660.
8. Cerami E, Gao J, Dogrusoz U, Gross BE, Sumer SO, Aksoy BA, Jacobsen A, Byrne CJ, Heuer ML, Larsson E *et al*: **The cBio cancer genomics portal: an open platform for exploring multidimensional cancer genomics data**. *Cancer discovery* 2012, **2**(5):401-404.
9. Gao J, Aksoy BA, Dogrusoz U, Dresdner G, Gross B, Sumer SO, Sun Y, Jacobsen A, Sinha R, Larsson E *et al*: **Integrative analysis of complex cancer genomics and clinical profiles using the cBioPortal**. *Science signaling* 2013, **6**(269):pl1.
10. Prat A, Karginova O, Parker JS, Fan C, He X, Bixby L, Harrell JC, Roman E, Adamo B, Troester M *et al*: **Characterization of cell lines derived from breast cancers and normal mammary tissues for the study of the intrinsic molecular subtypes**. *Breast Cancer Res Treat* 2013, **142**(2):237-255.
11. Hellemans J, Mortier G, De Paepe A, Speleman F, Vandesompele J: **qBase relative quantification framework and software for management and automated analysis of real-time quantitative PCR data**. *Genome Biol* 2007, **8**(2):R19.
12. Pfaffl MW: **A new mathematical model for relative quantification in real-time RT-PCR**. *Nucleic Acids Res* 2001, **29**(9):e45.
13. Benjamini Y, Hochberg Y: **Controlling the false discovery rate: a practical and powerful approach to multiple testing**. *Journal of the Royal Statistical Society Series B (Methodological)* 1995, **57**(1):289-300.
14. McDonald JH: **Handbook of Biological Statistics (3rd ed.)**. Baltimore, Maryland: Sparky House Publishing; 2014.

Additional Table 1. Clonal cell lines used in qPCR panel.

	Isoform 1	Isoform 2	Isoform 3	Isoform 4	Isoform 5
**Parental line**	**T47D-pHUSH-ELF5-Isoform 1-V5 (pool)**	**T47D-pHUSH-ELF5- Isoform 2-V5 (pool)**	**T47D-pHUSH-ELF5- Isoform 3-V5 (pool)**		
**T47D clones *qPCR round 1***	T47D-pHUSH-ELF5-Isoform 1-V5 Clone 2*	T47D-pHUSH-ELF5- Isoform 2-V5 Clone 8*	T47D-pHUSH-ELF5- Isoform 3-V5 Clone 10	Not tested	Not tested
	T47D-pHUSH-ELF5- Isoform 1-V5 Clone 10	T47D-pHUSH-ELF5- Isoform 2-V5 Clone 9	T47D-pHUSH-ELF5- Isoform 3-V5 Clone 11		
**T47D clones *qPCR round 2***	T47D-pHUSH-ELF5- Isoform 1-V5 Clone 9	T47D-pHUSH-ELF5- Isoform 2-V5 Clone 13	T47D-pHUSH-ELF5- Isoform 3-V5 Clone 20*		
	T47D-pHUSH-ELF5- Isoform 1-V5 Clone 16		T47D-pHUSH-ELF5- Isoform 3-V5 Clone 26		
**Parental line**		**MDA-MB-231-pHUSH-ELF5-TV2-V5 (pool)**	**MDA-MB-231-pHUSH-ELF5-Isoform 3-V5 (pool)**		
**MDA-MB-231 clones *qPCR round 1***	Not tested	MDA-MB-231-pHUSH-ELF5-Isoform 2-V5 Clone 1	MDA-MB-231-pHUSH-ELF5-Isoform 3-V5 Clone 2*	Not tested	Not tested
		MDA-MB-231-pHUSH-ELF5-Isoform 2-V5 Clone 7*	MDA-MB-231-pHUSH-ELF5-Isoform 3-V5 Clone 7		
**MDA-MB-231 clones *qPCR round 2***		MDA-MB-231-pHUSH-ELF5-Isoform 2-V5 Clone 6	MDA-MB-231-pHUSH-ELF5-Isoform3-V5 Clone 20		
			MDA-MB-231-pHUSH-ELF5-Isoform 3-V5 Clone 22		

* Also used in timecourse experiment (figure 6)

Additional Table 1. Clonal cell lines used in qPCR panel. All clonal lines were derived from a parental line as listed. Clones were either used in round 1 (116 genes) or round 2 (27 genes). Asterisk indicates that line was also used in the time course experiment shown in Figure 6.

Additional Figure 1

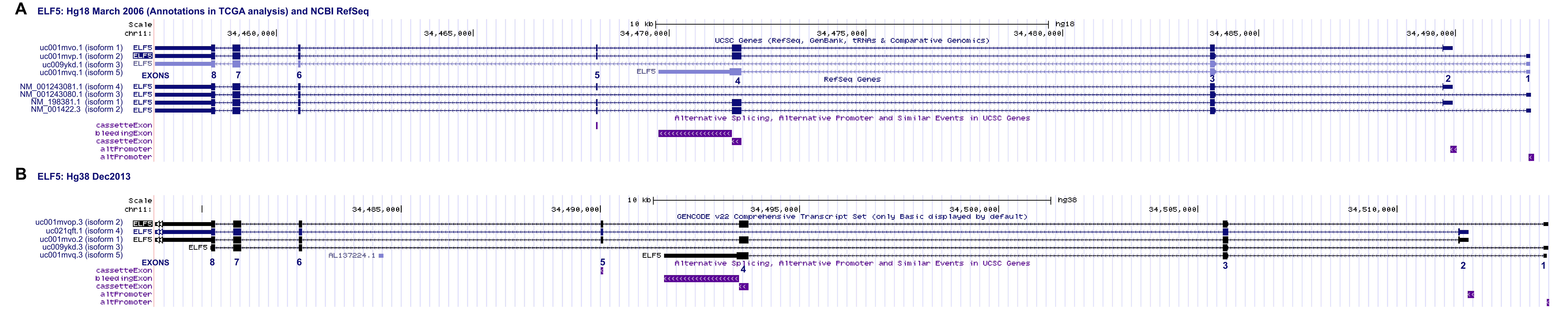

Additional Figure 2

A

	Mean Percentage				Median Percentage			
	TV1	TV2	TV3	Other	TV1	TV2	TV3	Other
Bladder	0.2%	68.1%	24.0%	7.8%	0.0%	86.9%	11.5%	0.9%
Head/Neck	1.8%	90.6%	6.5%	1.0%	0.0%	95.2%	0.0%	0.8%
Kidney	86.3%	9.4%	2.3%	2.1%	91.8%	3.9%	0.9%	1.8%
Lung	14.1%	81.1%	3.3%	1.5%	13.9%	82.1%	0.0%	1.2%
Prostate	3.2%	82.8%	12.3%	1.7%	0.0%	89.3%	1.4%	1.3%

B

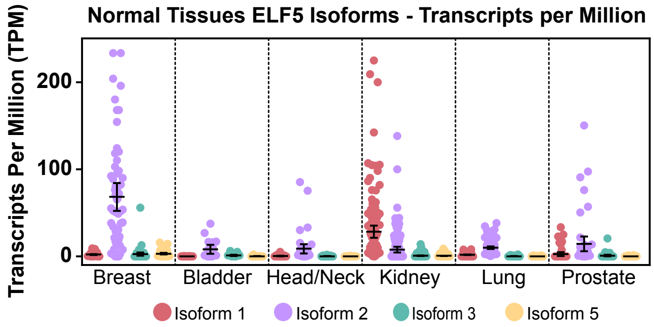

C

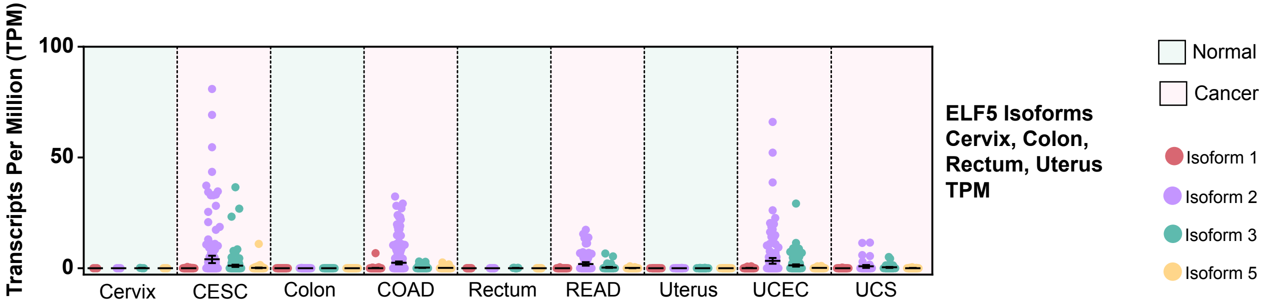

D

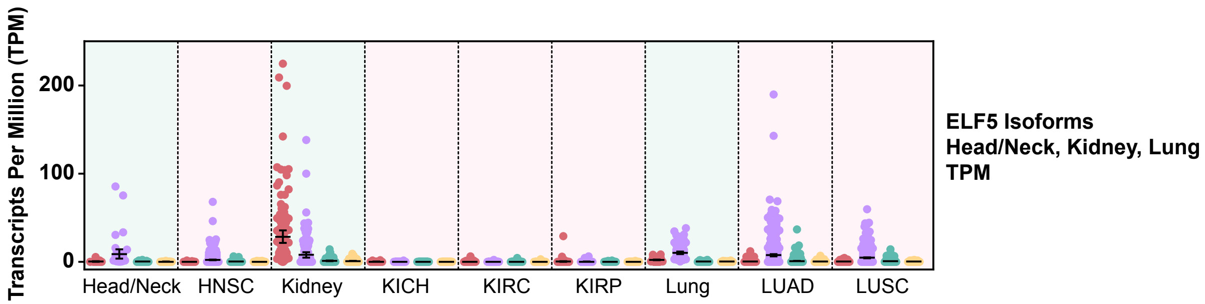

E

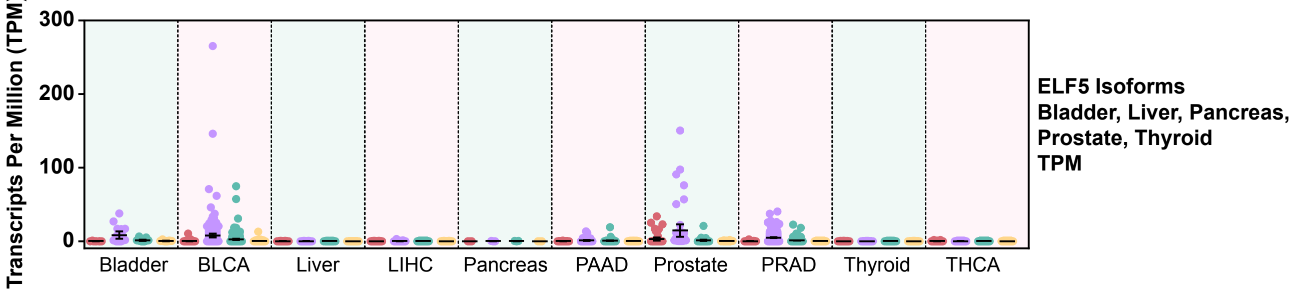

F

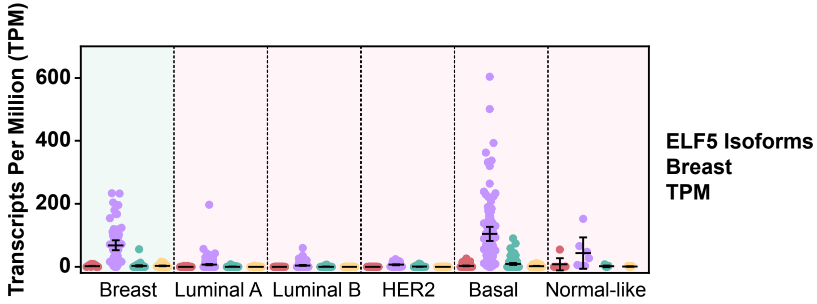

Additional Figure 3

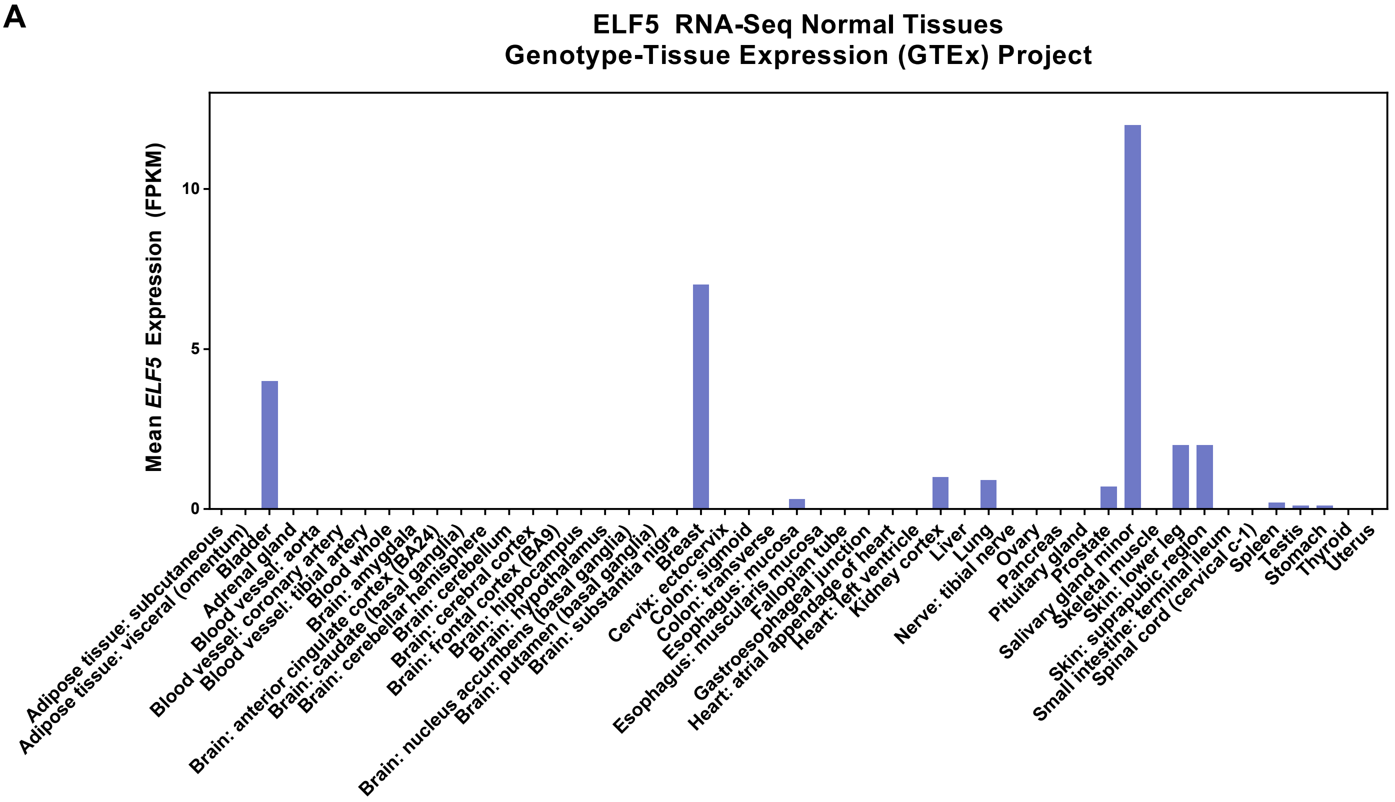

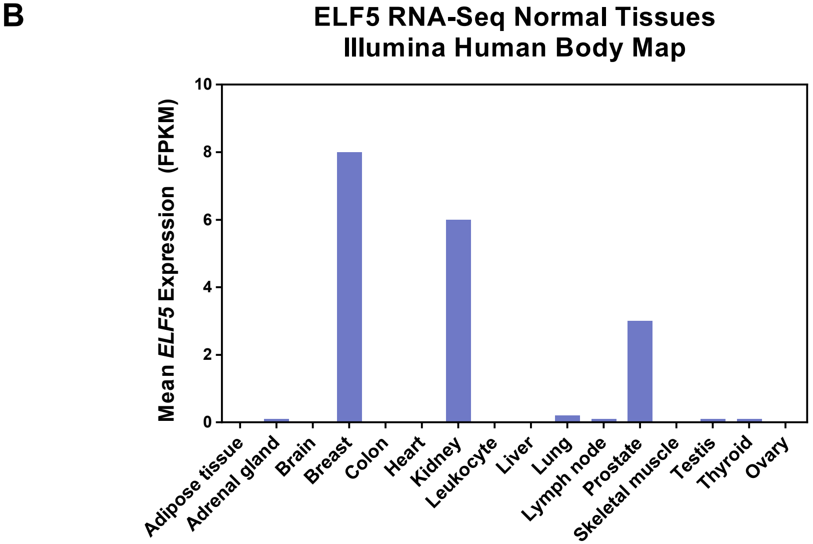

Additional Figure 4

A EdgeR differential expression analysis

	CESC	COAD	READ	UCEC	UCS	BRCA	HNSC	KICH	KIRC	KIRP	LUAD	LUSC	PRAD	BLCA	LIHC	PAAD	THCA	Assorted
Fold change	293.5	300.3	65.6	265.7	Filt.	0.45	0.17	0.005	0.004	0.02	0.63	0.40	0.24	1.08	Filt.	54.3	Filt.	NA (no normal samples)
FDR	0.13	5.9E-17	0.001	5.1 E-14		0.0003	2.1E-15	9.7 E-52	1.1 E-248	1.0 E-42	0.010	4.9 E-8	1.9E-19	0.94		0.29		

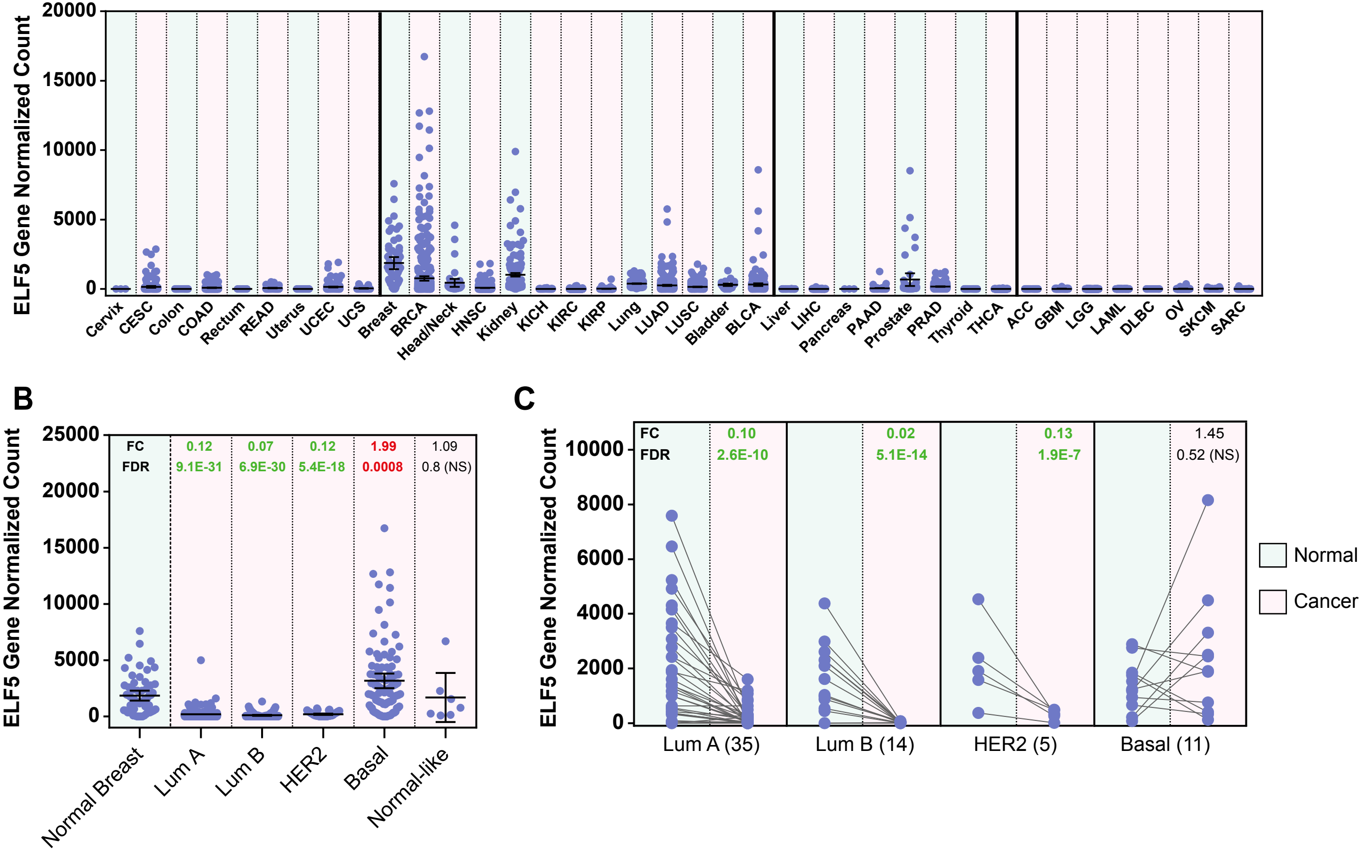

Additional Figure 5

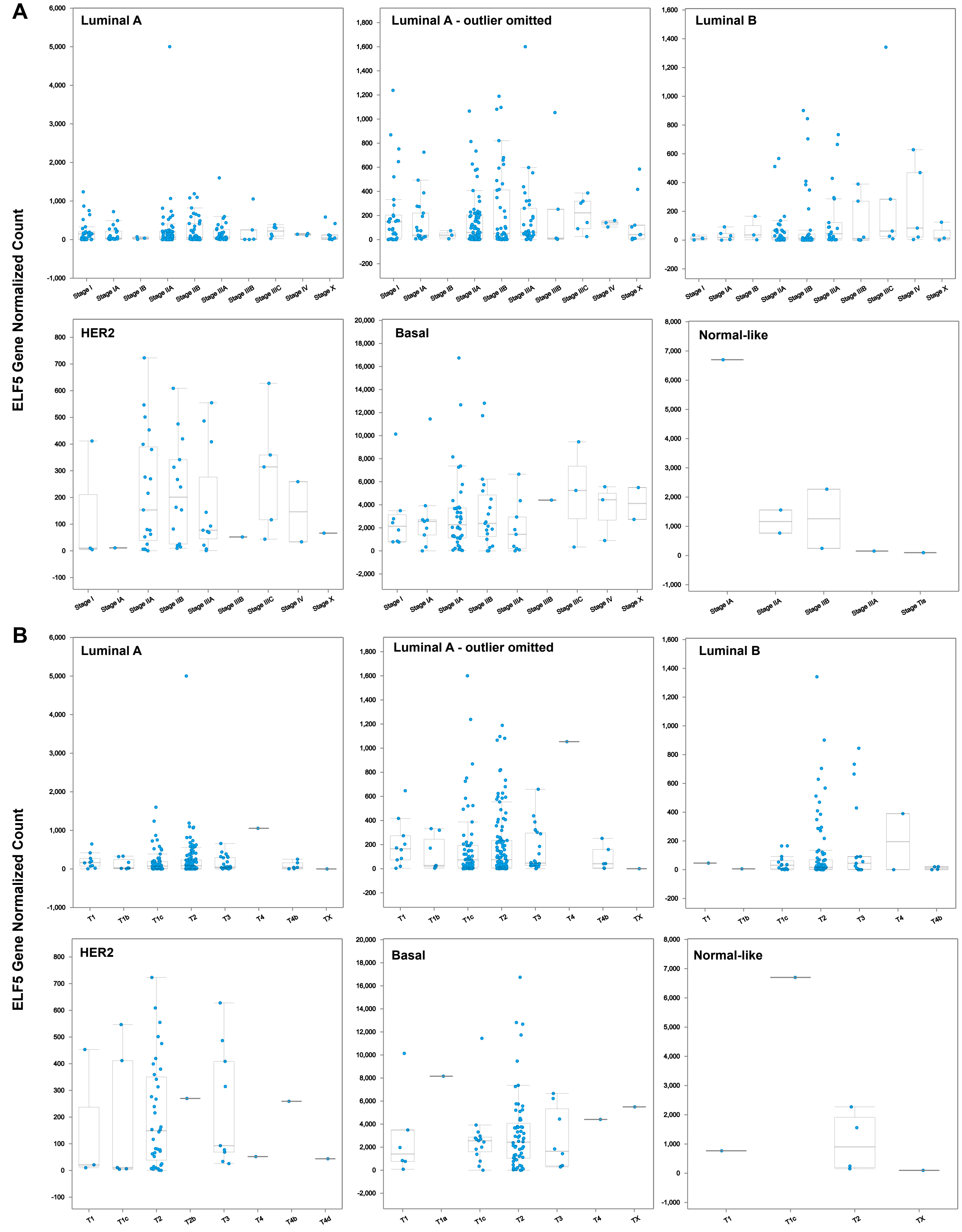

Additional Figure 6

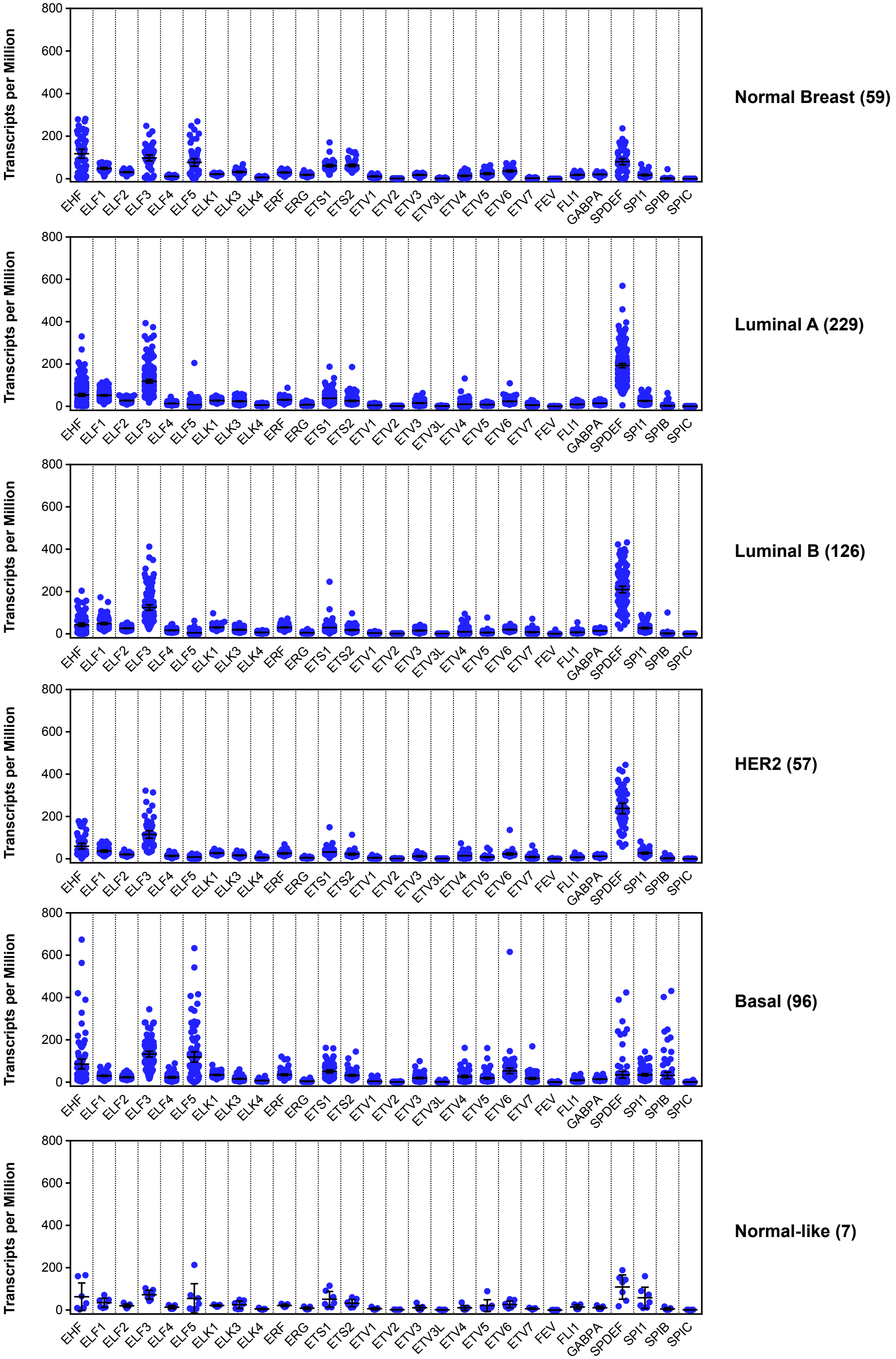

Additional Figure 7

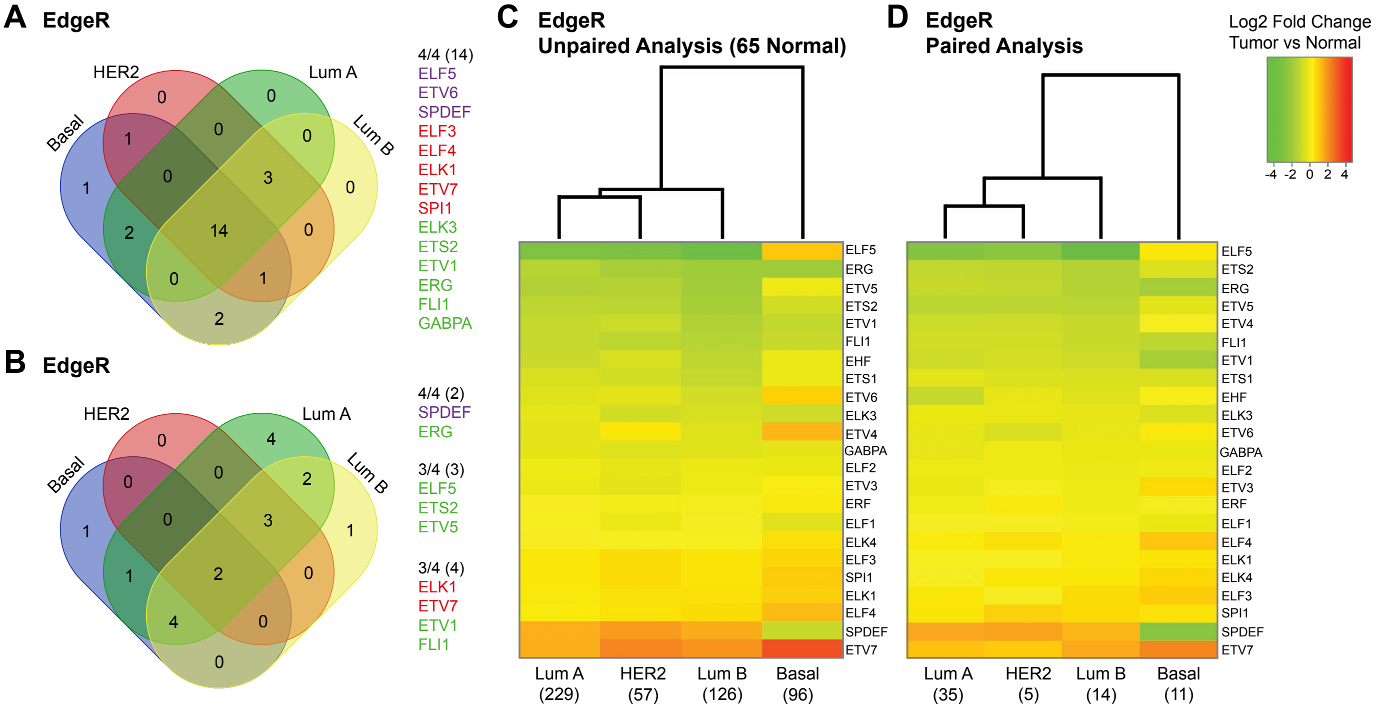

Additional Figure 8

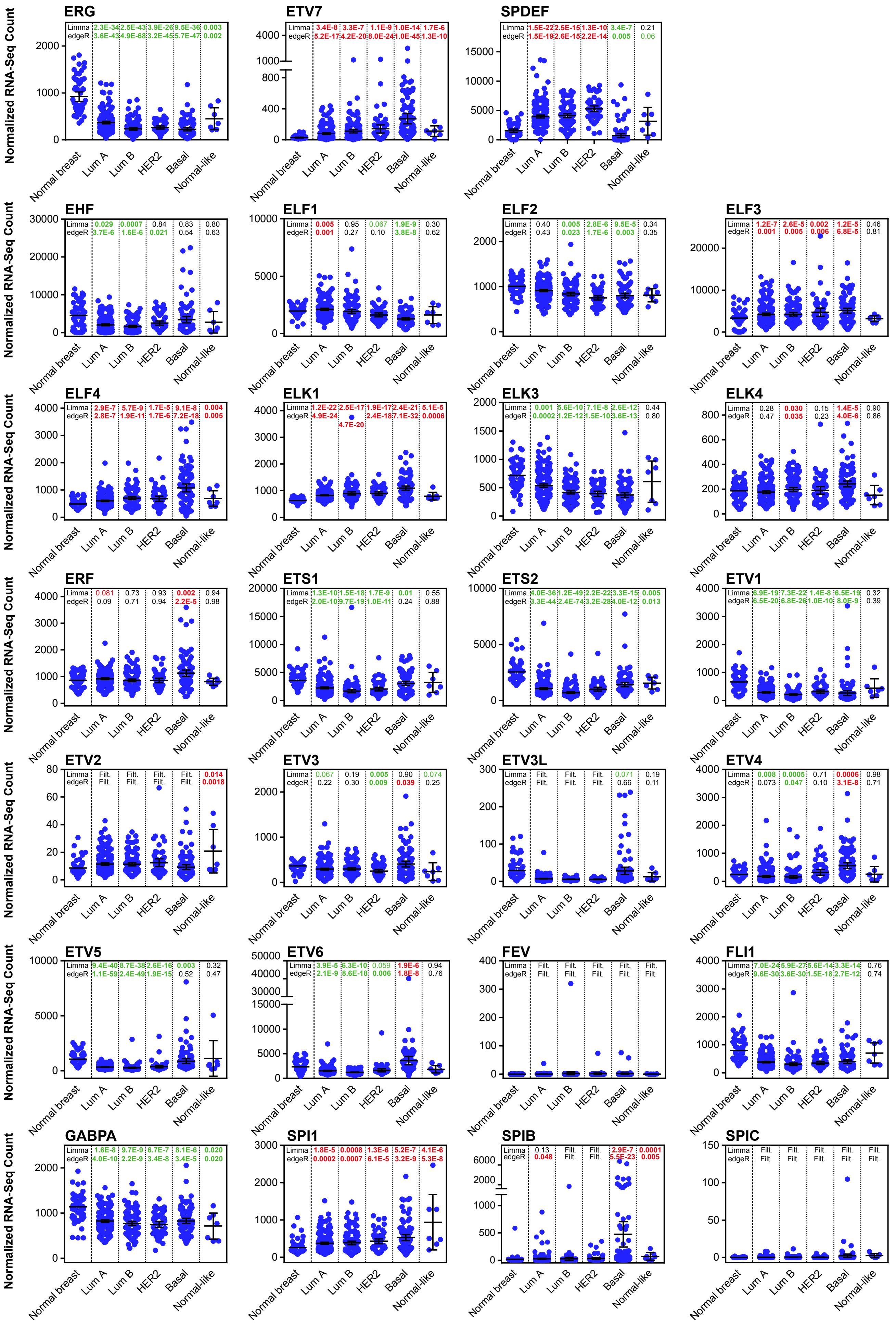

Additional Figure 9

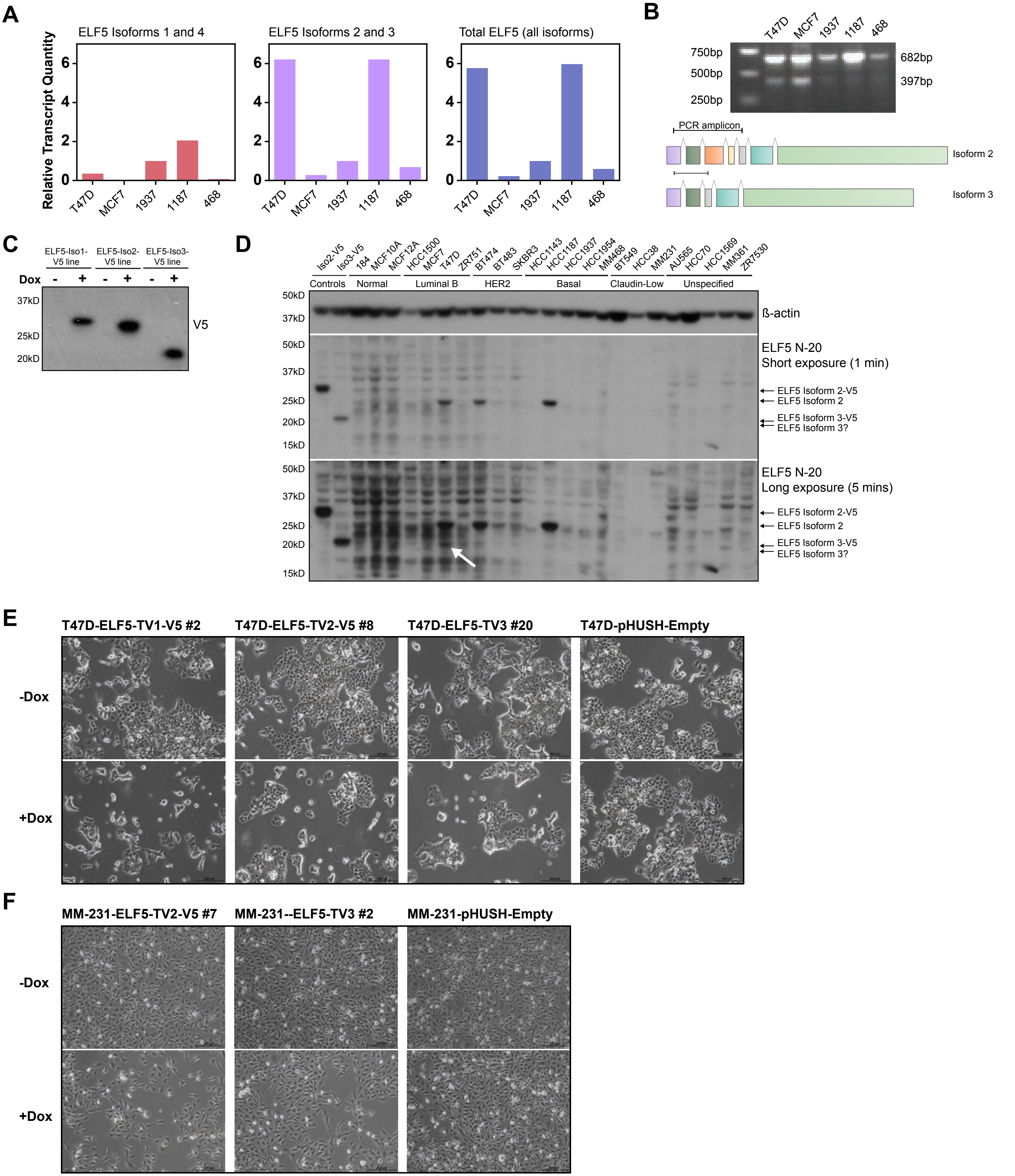

Additional Figure 10

qPCR Workflow

1

Assay design - Roche ProbeFinder
Criteria (if possible):
Intron-spanning
Detects all transcript variants
Unique primers
Short amplicon

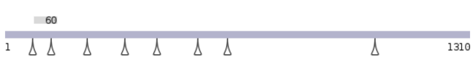

Use Universal ProbeLibrary probe: #60, cat.no. 04688589001

Primer	Length	Position	Tm	%GC	Sequence
Left Primer	19	83 - 101	60	58	agccacatcgctcagacac
Right Primer	19	130 - 148	60	53	gcccaatacgaccaaatcc
Amplicon (66 nt)					
agccacatcgctcagacaccatggggaaggtgaaggtcggagtcaacggatttggtcgta ttgggc					

2

Assay test
6-point dilution series to
create standard curve
Efficiency = 10^(-1/slope)-1
Ideally 0.90-1.1

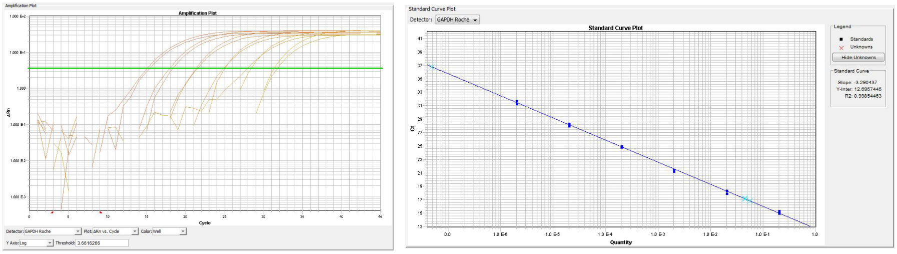

3

Experimental design
Standard curve for every assay
Negative controls - no template,
no reverse transcriptase and water
2-3 technical replicates per sample
2-4 clones (-/+dox) per cell line group

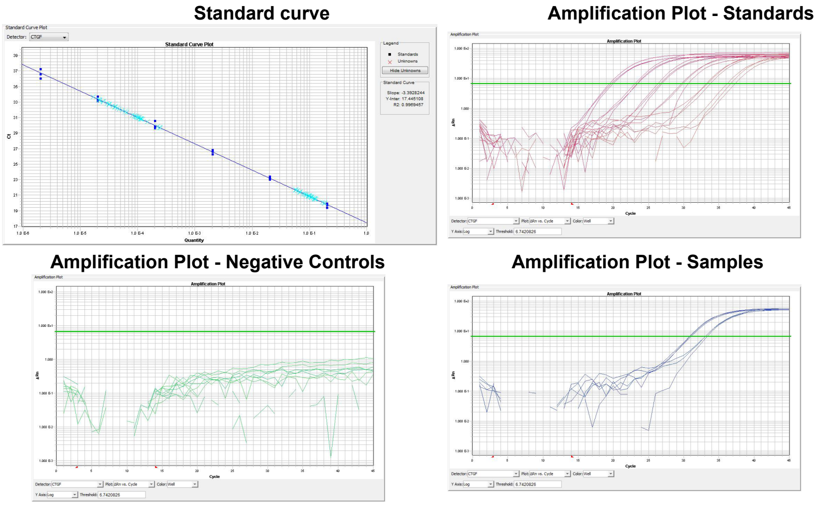

4

Analysis of results
SDS 2.4 software
qbase+ software (Biogazelle)
Quality control
Standard curve quantity calculation
Normalization to reference gene (GAPDH)
Standard curve samples as inter-run calibrators
for assays repeated in round 2
Statistical analysis

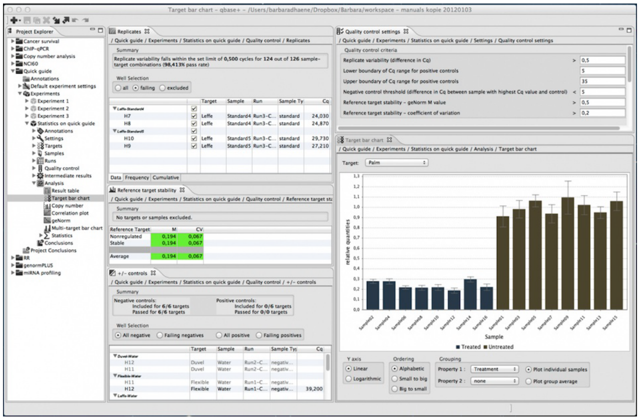

ADDITIONAL FIGURE LEGENDS

Additional Figure 1. UCSC genome browser screenshot with annotations showing (A) *ELF5* transcripts in Hg18 March 2006 and NCBI RefSeq and (B) the most recent transcripts in Hg38 December 2013. Hg18 transcript names match those that appear in The Cancer Genome Atlas RNA-sequencing analysis files. The more recent Hg38 includes an equivalent for NCBI *ELF5* Isoform 4 that does not appear in Hg18.

Additional Figure 2. Additional *ELF5* TCGA RNA-Sequencing data for normal and cancer samples. (A) Mean (left) and median (right) percentage values for *ELF5* isoforms in selected normal tissues. (B) Equivalent to graph shown in Figure 2B using 'Transcripts per million' (TPM) values instead of quantile normalized RNA-Seq counts. Plotted values represent individual samples and error bars show the mean with 95% confidence interval. (C-F) Equivalent to graphs shown in Figure 3B-E and Figure 4A using TPM values instead of quantile normalized counts. Plotted values represent individual samples and error bars show the mean with 95% confidence interval.

Additional Figure 3. Additional RNA-Seq datasets for normal tissues. (A) Mean *ELF5* FPKM values for 49 normal tissues from the Genotype-Tissue Expression (GTEx) Project. (B) Mean *ELF5* FPKM values for 16 normal tissues from the Illumina Human Body Map.

Additional Figure 4. *ELF5* expression is significantly altered in cancer - results from edgeR differential expression analysis. All fold change (FC) and False Discovery rate (FDR) are from edgeR (instead of Limma voom) differential expression analysis, with green values in bold indicating a significant downregulation and red values in bold a significant upregulation compared to normal (FDR<0.05). Filt. indicates gene filtered from edgeR analysis due to low expression. (A) *ELF5* gene expression (quantile normalized counts) for selected normal tissues and cancers with edgeR FC and FDR values. (B) *ELF5* gene expression (quantile normalized counts) for normal breast and breast cancer

subtypes, as a comparison for Figure 4A. (C) *ELF5* gene expression (quantile normalized counts) for patients with matched normal and cancer samples with edgeR FC and FDR values. Numbers in parentheses indicate sample pairs per group.

Additional Figure 5. Stage compared to *ELF5* expression in breast cancer subtypes. (A) *ELF5* gene expression (quantile normalized counts) for each breast cancer subtype plotted against American Joint Committee on Cancer (AJCC) stage. Plotted values represent TCGA RNA-sequencing samples. (B) *ELF5* gene expression (quantile normalized counts) for each breast cancer subtype plotted against AJCC tumor stage. Plotted values represent TCGA RNA-sequencing samples.

Additional Figure 6. ETS family gene expression in normal breast and breast cancer subtypes (TPM). Values are shown as 'Transcripts per million' (TPM), corrected for transcript length and allowing for limited comparison of expression within sample groups. Plotted values represent individual samples and error bars show the mean with 95% confidence interval. Numbers in parentheses after graph titles show the number of samples per group.

Additional Figure 7. Expression of other ETS family members is also altered in breast cancer, with the basal subtype having a distinct ETS expression profile - results from edgeR differential expression analysis. TCGA RNA-Seq edgeR differential expression analysis data for ETS family members, using results from edgeR (instead of Limma voom) differential expression analysis. (A) Venn diagram showing number of ETS family members significantly altered in breast cancer subtypes compared to normal (FDR<0.05). All subtypes were compared to a common set of 65 normal samples (unpaired analysis). Genes altered in all 4 subtypes are listed (red = upregulation, green = downregulation, purple = differentially regulated in basal subtype compared to other subtypes). (B) Venn diagram showing number of ETS family members significantly altered in breast cancer subtypes compared to normal (FDR<0.05), using paired normal and tumor samples from the same patient. Genes

altered in at least 3 of 4 subtypes are listed, with color-coding as above. (C) Clustered heat map of ETS factor edgeR log2 fold change, comparing tumor samples to 65 normal samples. Legend is shown next to panel D. Rows are sorted by Luminal B values (smallest to largest) and columns are sorted according to clustering. Numbers in parentheses are samples per group. (D) Clustered heat map of ETS factor edgeR log2 fold change, comparing paired normal and tumor samples, with sorting as above. Numbers in parentheses are sample pairs per group.

Additional Figure 8. ETS family expression gene expression in normal breast and breast cancer subtypes (Normalized Counts). Values are shown as quantile normalized RNA-seq counts, allowing for comparison across subtypes but not correcting for transcript length. Plotted values represent individual samples and error bars show the mean with 95% confidence interval. False Discovery rate (FDR) from unpaired Limma voom (top) and edgeR (bottom) differential expression analysis are shown, with bold green indicating a significant downregulation and bold red a significant upregulation compared to normal (FDR<0.05). Non-bold red or green values indicate FDR 0.05-0.10.

Additional Figure 9. Phenotype of pHUSH-ELF5-V5 breast cancer clonal cell lines. (A) qPCR for breast cancer cell lines showing relative levels of *ELF5* isoform pairs 1/4 and 2/3 and total *ELF5* (all isoforms). Single experiment with values normalized to HCC1937 samples. Abbreviations: 1937 = HCC1937, 1187 = HCC1187, 468 = MDA-MB-468. MDA-MB-231 cells have undetectable *ELF5*. (B) End-point PCR designed to amplify Isoforms 2 and 3 simultaneously in same panel of cell lines. DNA gel shows amplicons present after 25 PCR cycles. (C) V5 western blot of cell lines overexpressing ELF5 Isoform 1, 2 or 3 (tagged with V5) on addition of doxycycline (Dox), demonstrating relative isoform sizes. (D) Western blot for endogenous ELF5 in a panel of breast cancer cell lines, classified by molecular subtype. Controls in lanes 1 and 2 are cell lines overexpressing ELF5 Isoform 2 or 3 (tagged with V5) on addition of doxycycline (Dox). A possible ELF5 Isoform 3 band in HCC1187 cells is marked with a

white arrow. (E-F) Light microscope images taken at day 4 for T47D clonal cell lines (E) and MDA-MB-231 cell lines (F), treated with vehicle (top row) or doxycycline (bottom row).

Additional Figure 10. qPCR workflow. Details of qPCR experiment, including assay design, testing, experimental design and results analysis.
